# Advanced Chemophenetic Analysis of Essential Oil from Leaves of *Piper gaudichaudianum* Kunth (Piperaceae) Using a New Reduction-Oxidation Index to Explore Seasonal and Circadian Rhythms

**DOI:** 10.3390/plants10102116

**Published:** 2021-10-06

**Authors:** Ygor Jessé Ramos, Claudete da Costa-Oliveira, Irene Candido-Fonseca, George Azevedo de Queiroz, Elsie Franklin Guimarães, Anna C. Antunes e Defaveri, Nicholas John Sadgrove, Davyson de Lima Moreira

**Affiliations:** 1 Instituto de Biologia, Pós-Graduação em Biologia Vegetal, Universidade do Estado do Rio de Janeiro, Maracanã, Rio de Janeiro 20550-013, Brazil; ygorjesse@gmail.com; 2 Diretoria de Pesquisa do Instituto de Pesquisas Jardim Botânico do Rio de Janeiro, Jardim Botânico, Rio de Janeiro 22460-030, Brazil; elsie.guimaraes@jbrj.gov.br; 3 Centro de Responsabilidade Socioambiental do Instituto de Pesquisas Jardim Botânico do Rio de Janeiro, Jardim Botânico, Rio de Janeiro 22460-030, Brazil; georgeazevedo08@gmail.com (G.A.d.Q.); anna.defaveri@gmail.com (A.C.A.e.D.); 4 Fundação Oswaldo Cruz, Farmanguinhos, Manguinhos, Rio de Janeiro 21041-250, Brazil; deteoliveira@hotmail.com (C.d.C.-O.); irene.fonseca2903@gmail.com (I.C.-F.); 5 Jodrell Science Laboratory, Royal Botanic Gardens Kew, Richmond TW9 3DS, UK; n.sadgrove@kew.org

**Keywords:** *Piper*, medicinal plant, terpenoids, chemodiversity, chemophenetic

## Abstract

The aromatic species *Piper gaudichaudianum* Kunth (Piperaceae) is widely used in Brazil for medicinal and ritualistic applications. In the current study, chemophenetic patterns were realized across season and circadian rhythm based on the chemical profile of essential oils (EOs) from leaves. Hydrodistilled essential oils were analyzed by GC-MS and GC-FID, and a new calculation of metabolite oxidation level, averaged for each individual molecule component of the EO, was used to explore the patterns of metabolism/biosynthesis. This new index used an intermediate calculation, the ‘weighted average redox standard’ (S_RO_), to enable a value for mixtures of metabolites to be generated, the ‘general mixture redox index’ (GM_OR_). The indices were subjected to a proof-of-concept approach by making comparison to outcomes from multivariate analyses, i.e., PCA and HCA. Chemical analysis demonstrated that the essential oils were dominated by sesquiterpenes, constructed of 15 classes of compound (C-skeletons), and 4 C-skeletons were recognized in the monoterpene group, giving a total of 19. The variation of chemical profiles was distinct at different phenological stages, but stronger chemical variation was evident between day and night as compared to season. Furthermore, due to comprehensive sampling across different regions, nine chemotypes were recognized, including those previously reported. The S_RO_ and GM_RO_ indices demonstrate that phenological variation of chemistry is mainly an outcome of redox fluctuations in terpene biosynthesis, changing from day to night. These indices also corroborate that chemical diversity is increased with oxidative metabolism. Lastly, the current study demonstrates pronounced phenotypic plasticity in *P. gaudichaudianum*, which makes it a suitable candidate to help further our understanding of chemophenetics and chemical ecology.

## 1. Introduction

*Piper gaudichaudianum* Kunth (Sin. *Artanthe gaudichaudiana* (Kunth) Miq.; *Piper obscurum* C.DC.) is a native South American member of Piperaceae that is widely distributed in Brazil, mainly in the Atlantic Forest [1,2]. The fruit from this ecologically important species is a source of nutrition for fruit bats [3,4] and birds [4,5]. Furthermore, in humid forests, both the leaves and fruit are eaten by a diversity of insects [6,7,8,9,10]. This species also participates in various biotic interactions [11,12,13]. Fruit from *P. gaudichaudianum* is more than a mere food source, as it plays a significant role in determining reproductive success and population density of several species of Brazilian fruit bat that live in the Atlantic Forest [3,14,15,16,17,18,19,20,21].

The colloquial names given to *P. gaudichaudianum* by the Brazilian people include “Jaborandi”, “Falso-jaborandi” and “Pariparoba”. The first written record of use of this species as a medicinal plant is from the mid-19th century [22]. Ethnobotanical surveys describe how infusions, or the chewing of fresh leaves, provides relief of dental pain. Furthermore, the tea from leaves is used as a cholagogue (stimulator of bile secretion) and a digestive aid. Anecdotal accounts also describe *P. gaudichaudianum* as an adjuvant against tumors, joint pain, musculoskeletal diseases and against infectious conditions, i.e., infections of the skin, ears, nose and oropharynx [23,24,25,26]. The species is also known for its aromatic character, causing it to become an adulterant to *Pilocarpus jaborandi* Holmes (Rutaceae), a commercial species used in the informal medicinal species market [27]. Nevertheless, in the compendium of “Florais de Saint Germain”, the essence or aroma of *P. gaudichaudianum,* is prescribed for “relaxation” and the reduction of “mental rigidity” [28]. This species is also used under the name “Ìyèyé” for ritualistic practice in Afro-Brazilian religions, either as a smoke or as an infusion in a bath or fermented drink, to imbibe the qualities of the warrior deity “Orixá Xangô” (i.e., Shango) [29,30,31]. It is worth noting that for the rituals of this religion, there is a specification that the leaves of *P. gaudichaudianum* are harvested between 12 p.m. and 6 p.m. [31]. If these requirements were determined from tangible empiricism, then this provides the impetus to investigate a chemical correlation to such diurnal specificities. Previous phytochemical studies have described various chemical components, such as terpene alcohols, phytosterols, vitamin E (tocopherols), fatty acids, triterpenes, flavonoids, alkaloids, chromenes and prenylated derivatives of benzoic acid [11,32,33,34,35,36,37]. The aromatic character of the species has been investigated in previous phytochemical studies that show this species to yield a moderate quantity of essential oil (EO). The EOs of *P. gaudichaudianum* comprise a high percentage of monoterpenes, sesquiterpenes and arylpropanoids, with relative amounts depending on the collection site and duration of hydrodistillation [38,39,40,41,42,43,44,45,46].

Studies have also corroborated the biological effects from the extracts, such as positive outcomes in the context of antifungal, antibacterial, insecticidal, larvicidal, analgesic, anti-inflammatory, antileishmanial and antituberculosis activities [39,41,47,48,49,50,51,52]. Although research on this species has been comprehensive, there are no approaches with the purpose of analyzing the phenotypic plasticity of chemical components, such as volatile organic compounds. In other species, the influence of biotic and abiotic factors on the composition of EOs is frequently reported in the literature [2,53,54,55]. Phenotypic plasticity has become a major challenge in the context of understanding correlators of chemistry to taxa [56,57]. Hence, tools/methodologies to evaluate and to interpret phenomena around phenoplasticity are still evolving [58,59,60]. Among the methods for assessing plasticity and chemotype at different spatial scales, there are α-, β- and γ-chemobiodiversity indices that were put forth during the era of chemotaxonomy, particularly the mid-20th century. The details of each of these indices are as follows: (1) α-chemobiodiversity indices, i.e., Shannon index [61,62,63,64], Simpson’s diversity index [61,65,66], Pielou’s uniformity index [67] and Iason’s chemodiversity index [65,68]; (2) β-chemodiversity indices, i.e., Sorensen index [61,69], Jaccard’s index [61,70] and Cody’s index [61,71]; (3) γ-chemodiversity indices, i.e., chemical similarity index [13], and indices of chemical difference in relative abundance—Rao index [72]. All these parameters favor the qualitative “absence or presence” response of compounds but neglect quantitative effects (where expression patterns change). Furthermore, the major shortcoming of these earlier approaches to chemophenetic study is that they do not use measures to predict the structural patterns of compounds [60]. Unfortunately, since chemophenetic approaches have not been updated in the last 50 years, the derivatives of common biosynthetic groups continue to be overlooked as flagging a closer relationship between taxa or chemotypes than derivatives across the different chemical classes.

In the mid- to late-20th century, chemophenetics was known under the previous classification as chemotaxonomy. This outdated philosophy was, at the time, interrogated for its ability to inform the evolutionary progress of taxa [73,74,75]. In earlier thought, the oxidation level of specialized metabolites was regarded as a correlator to the age of taxa, wherein older taxa expressed less oxidized derivatives. Hence, as new taxa emerged in the course of natural selection and species radiation, the respective metabolites increased in their degree of oxidation, said to be in response to increased concentrations of oxygen in the atmosphere [76]. With the phylogenetics revolution, this theory was eventually discredited, but the exploration of patterns in metabolite oxidation has, nevertheless, continued to demonstrate reproducible patterns in chemical ecology studies. The issue faced with measuring and comparing metabolite oxidation within and across taxa is that it is difficult to ascribe a statistic or numerical value to metabolites that vary not only in oxidation level but also by their biosynthetic pathways and relative expression levels (quantities or yields). Over 50 years ago, Hendrickson [77] developed a calculation which he called the summed oxidation number (N_OX_) that gave an oxidation index that is calculated by the summation of values ascribed to all bonds in the molecule [78]. To further understand the patterns of metabolite oxidation, Emerenciano et al. [79] developed an index derived from N_OX_ to assess the oxidative stages of terpenoids relative to their unoxidized biosynthetic precursor. This index was applied to chemical profiles of EOs’ by Sayuri et al. [80], but the results are not useful because of a lack of variation in calculated values (0–2). However, these earlier types of approaches declined to use quantitative data as a dependent variable and focused on the presence or absence of compounds in the taxa [79]. Obviously, this was because during the mid-1990s instrumentation that gave precision in values for absolute or relative quantification was relatively new or still under development. Since quantitative techniques have advanced greatly into the 21st century, a revision is now possible and necessary.

While there has been no input or updates on the index of oxidation of complex mixtures of compounds since the 1990s, the oxidation of secondary metabolites is now viewed in a different light, as compared to earlier thought [81]. While the works of Gottlieb et al. [73,74] were regarded as innovative at the time, the discipline of chemical ecology has entered a new paradigm [60], so it will benefit from a metric that recognizes biosynthetic relatedness and oxidation level collectively. New meaning may therefore be found by revisiting this concept with an open mind. Furthermore, with the exponential growth of metabolomic analyses [82], the opportunity to update this calculation presents itself. It is therefore necessary to develop an index that can describe the homogeneity and the reduction-oxidation (redox) pattern of a complex mixture for α, β and γ-chemodiversity assessments.

The current study aims to: (a) evaluate for the first time the chemical composition, seasonal variation and circadian rhythm of EOs from leaves of a natural population of *P. gaudichaudianum* in an area of Atlantic Forest in the city of Rio de Janeiro; (b) Develop and submit to a Proof of Concept (PoC) a new predictive methodology to assess the redox of complex mixtures of compounds using the Weighted Average Redox Standard (S_RO_) and the General Mixture Redox Index (GM_RO_); (c) Set the variation and chemophenetic patterns in time and space scales for *P. gaudichaudianum* based on the EO analysis.

## 2. Results and Discussion

The yield and chemical composition of the EOs obtained by hydrodistillation from the leaves of *P. gaudichaudianum* are shown in Table 1 and Table 2. Pearson’s correlation is given in Table 3.

### 2.1. Essential Oil Yields

The EOs showed a slightly yellow color, which yielded in the range of 0.02 to 0.23% (*w/w*) (Table 1 and Table 2). These values were higher compared to some of the results described in the literature for this species (0.01–0.10%) [37,41]. Higher yield values were published for samples from Santa Maria (Rio Grande do Sul, Brazil) that ranged from 1.32 to 1.61% [43]. Intermediate values were recorded for samples collected in Atalanta (Santa Catarina, Brazil) (0.24 to 0.46%) [83].

For the seasonal study, the highest yields were recorded for those EOs obtained in November (0.11%), December (0.14%) and January (0.12%). The study of the circadian rhythm showed that the highest EOs expression occurred at 6 a.m. (0.23%) in the rainy season (R) and at 12 p.m. (0.16%) in the dry season (D). There was a statistical difference between the averages throughout the day for each season (*p* < 0.0001), as well as between night and day (*p* = 0.0351). However, comparing the average yields between the dry and rainy season, there was no significant difference (*p* = 0.4833). In both seasons, the night period (9 a.m. to 6 a.m.) afforded the high values of EO yield.

Pearson’s correlation analyses (Table 3) showed that there was an inversely proportional correlation in the dry season with the relative humidity (r = −0.887; *p* = 0.003), temperature (r = −0.787; *p* = 0.020) and radiation (r = −0.862; p = 0.006) in the circadian study. It is known that plant species tend to show different patterns of qualitative plastic responses from an EOs perspective at the level of shading (light intensity), increase in temperature and relative humidity. For example, *Piper umbellatum* L. showed higher EOs yields when grown in the shade [84], while *Matricaria recutita* L. (Asteraceae) yielded the highest when under intense light conditions [85]. A study correlating the yields of EO of P. umbellatum and photosynthetic activities described that when cyophyte plants, such as *P. gaudichaudianum*, are subjected to high irradiance, chlorosis and necrosis usually occur with the photodegradation of chromopigments, leading to reduced photosynthesis and biomass production [86]. In addition to this mechanism, there is a decrease in EO accumulation through evaporation provided by increases in gas exchanges, temperature, stomatal conductance and the CO_2_ assimilation rate [55,84,87,88,89].

### 2.2. Chemical Profile of the Essential Oil

Ninety-seven (n = 97) constituents were identified by GC-MS, corresponding to an average of 96.3% (91.5–99.8%) and 92.8% (84.5–97.5%) of the EO in the seasonal (S) and circadian (C) studies (Table 1 and Table 2), respectively. The EOs were found to be rich in non-oxygenated sesquiterpenes (S: 37.9–81.5%; C: 48.3–78.0%), followed by oxygenated sesquiterpenes (S: 10.5–50.5%; C: 16.2–44.8%) and oxygenated monoterpenes (S: 0.0–17.0%; C: 0.0–3.4%). The main identified compounds were bicyclogermacrene (S: 11.2–23.2%; C: 10.2–28.5%), followed by E-caryophyllene (S: 3.1–11.2%; C: 1.3–22.7%) and eudesmadiene (cis-eudesma-6,11-diene) (S: 2.8–15.3%; C: 1.4–21.7%), in addition to the oxygenated sesquiterpenes E-nerolidol (S: 3.8–22.9%; C: 0.3–15.4%), α-cadinol (S: 1.2–11.2%; C: 0.2–19.4%) and spathulenol (S: 0.1–3.3%; C: 1.39–15.9%). Bicyclogermacrene has been reported as the main compound in the EOs of some species of Piper, for example, *P. aduncum* L. (20.9%) (Bernuci et al. 2016); *P. amalago* L. (27.9%) [90]; *P. arboreum Aubl*. (49.5%) [91]; *P. cernuum Vell*. (25.1%) [90]; and *P. manausense Yunck*. (41.0%) [92].

The first study with the EO of *P. gaudichaudianum* was carried out with a sample obtained in the municipality of Sapiranga, Rio Grande do Sul State, South of Brazil, and described a chemical composition rich in α-humulene (37.5%) [45]. However, in the same Brazilian State, the sesquiterpenes E-nerolidol (22.1–22.4%) and α-humulene (16.5–37.5%) were assigned in EO from a specimen from the municipality of Riozinho [42,44], whereas specimens from the municipality of Santa Maria yielded EOs characterised by the phenylpropanoid dillapiole (57.8–70.5%) [43]. In the State of Paraná (South of Brazil) in different sites of the municipality of Curitiba, the major compounds identified were longipinanol (19.1%) and 5-epi-7-epi-α-eudesmol (13.3%) [40], 1-epi-cubenol (24.2%), cadalene (33.7%), E-caryophyllene (17.8%) and β-pinene (13.2%) [93]. For the municipality of Antonina (Parana State), δ-cadinene (45.3%) [47] or germacrene B (21.5%) and δ-cadinene (9.4%) were the major components [94]. In Diamante do Norte (Parana State), the main compounds were E-caryophylene (7.3–7.4%), β-pinene (3.8–6.6%) and δ-cadinene (5.6–7.1%) [95]. In the municipality of Piraquara (Parana State), 1-epi-cubenol (25.1%) and eudesm-7 (11)-en-4-ol (28.4%) dominated. In addition, germacrene B (21.5%) and δ-cadinene (9.3%) were registered as the main constituents in the municipality of Araquari, State of Santa Catarina, in the South of Brazil [39]. Viridiflorol (27.5%) and aromadendrene (15.6%) were identified as the major compounds in specimens from the municipality of Porto Velho, State of Rondônia, in the North of Brazil [41], and lastly, α-selinene (16.6%) and α-humulene (13.3%) were the main components from samples in the municipality of São Paulo, in the State of São Paulo, Southeast of Brazil [38].

### 2.3. Seasonal Variation of the Essential Oil

In the seasonal study, despite the relatively uniform distribution recorded throughout the year for non-oxygenated sesquiterpenes (Table 1 and Table 3, supplementary Table S1), in periods with greater precipitation (r = 0.701; *p*= 0.011) and relative humidity (r = 0.735; *p*= 0.006) there was an increase in non-oxygenated monoterpenes. The non-oxygenated sesquiterpenes showed correlations inversely proportional with precipitation (r = −0.591, p= 0.043) and directly to the temperature (r = 0.625, *p*= 0.030). The increase in the average monthly precipitation led to an increase in the concentration of oxygenated sesquiterpenes, a result confirmed by the significant value found in the correlations (r = −0.828; *p*= 0.001) (Table 3). The EOs from the aerial parts of Peperomia galioides Kunth (Piperaceae) showed similar increases in the relative percentages of oxygenated sesquiterpenes in the period of greatest precipitation [96]. Some studies persuade the hypothesis that plant species create mechanisms to control the biosynthetic route in proportion to the available resources, hence, in the current study, the high water content in the environment triggers a biosynthetic change that is more resource taxing [97,98,99].

**Figure 1:**
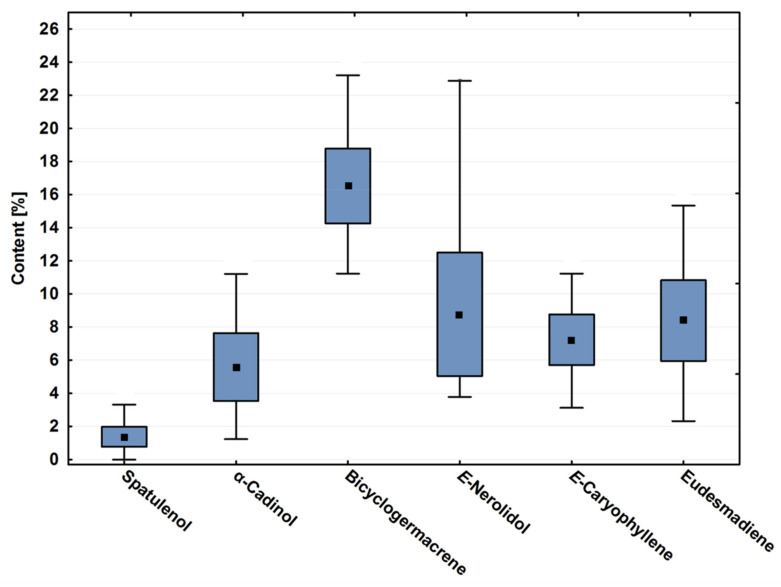
Box plot analyses of the major compounds (%) registered in the essential oils from leaves of *Piper gaudichaudianum* Kunth (Piperaceae) collected monthly for the seasonality study (January to December 2017). Means followed by different letters are significantly different according to Tukey test (*p* < 0.05).

Bicyclogermacrene, E-caryophyllene, eudesmadiene, E-nerolidol and α-cadinol contents showed significant variation throughout the year (*p* < 0.01). Figure 1 is a box plot that demonstrates these variations of these major compounds throughout the season. It was possible to observe that, even showing high annual variation, the average of bicyclogermacrene differs from the other major compounds (*p* < 0.01). The oxygenated sesquiterpene E-nerolidol showed the greatest variation in content (Table 1, Figure 1).

During 2017, P. gaudichaudianum reproductive organs matured in the period when the average rainfall increased, just after the dry period, in the months of January (infructecences and inflorescences) to February (infructecences) and early November (inflorescences) to December (infructecences and inflorescences). It is described in the literature that reproductive phenophases occur mainly in the rainy season [100]. Interestingly, in P. gaudichaudianum the relative percentage of E-nerolidol increases by up to four times in periods of higher rainfall after the dry period. When testing this hypothesis, we observed directly proportional and significant values in Pearson’s correlation between the content of E-nerolidol with the precipitation (r = 0.769; p = 0.003) and relative humidity (r = 0.791; *p* = 0.002).

**Figure 2:**
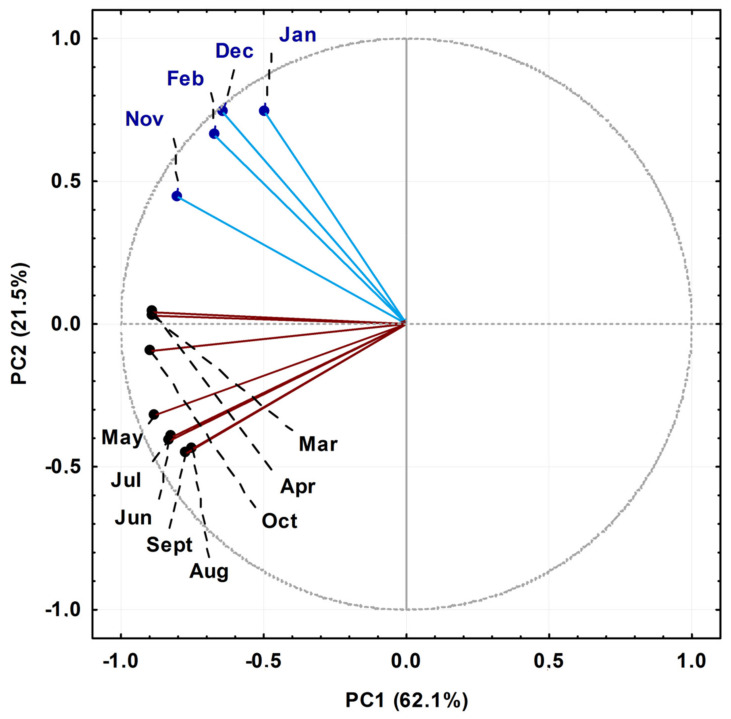
Biplot (Principal Component Analysis—PCA) resulting from the analysis of the essential oils obtained from leaves of *Piper gaudichaudianum* Kunth (Piperaceae) collected for the seasonality study monthly, from January to December 2017.

The EO components of *P. gaudichaudianum* with relative percentages greater than 5% were submitted for statistical analysis. The Principal Component Analysis (PCA) of the seasonal study showed that the main components PC1 (62.1%) and PC2 (21.5%) explained 83.6% of the total chemical variation between all samples, which were classified into two groups, as shown in Figure 2. The loading plot (not shown) demonstrated that bicyclogermacrene in PC1 (−8.6) was a negative contributor, and in PC2 (+0.7) it was a positive contributor. For E-nerolidol, in PC2 (+5.1) it was a positive contributor for samples collected in January, February, November and December, and in PC1 (−1.7) it was a negative contributor for samples collected from March to October. Lastly, the compounds eudesmadiene, α-cadinol and E-caryophyllene also contributed, albeit minor, to the PCA grouping pattern. The samples collected in the months of April, March and October, considered transition months between seasons in the South Hemisphere, were characterised by variations to the concentration of E-nerolidol. That said, group I (January, February, November and December) was characterised as high in bicyclogermacrene and E-nerolidol, and group II (March to October) was also rich in bicyclogermacrene but had higher expression levels of E-caryophyllene > eudesmadiene > α-cadinol.

**Figure 3:**
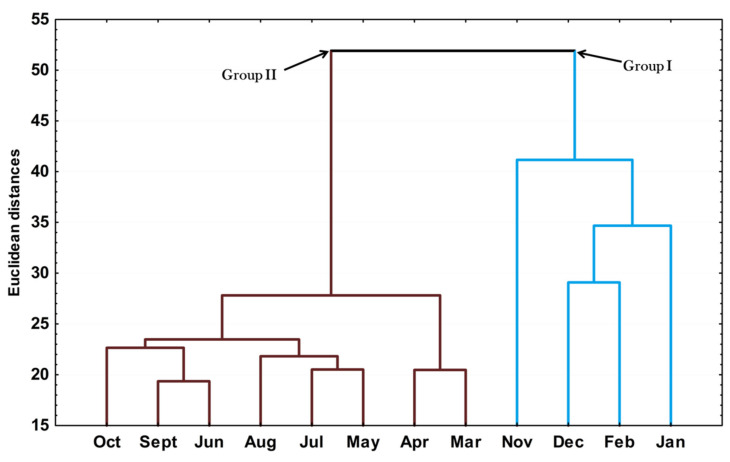
Dendrogram representing the similarity relation of the essential oils composition from leaves *Piper gaudichaudianum* Kunth (Piperaceae) collected for the seasonality study monthly, from January to December 2017.

The hierarchical cluster analysis (HCA) on the essential oils from different seasons is provided in Figure 3. The clusters in this dendrogram corroborated the results found in the PCA analysis, i.e., the samples were grouped into two clusters (Groups I and II). Interestingly, the clustering of group 1 agreed with the months when the species was in the reproductive phase and reflected the importance of E-nerolidol in this process.

The phenological influence on the chemical profiles of EOs from leaves of *P. gaudichaudianum* has already been reported in several species [2,101,102,103]. It is known that the expression levels of EOs depend on physiological resource allocation patterns that are established in response to abiotic factors to ensure that the conditions for growth, defense and/or reproduction are met [2,104]. Piper mollicomum Kunth, for example, showed high amounts of the oxygenated monoterpene linalool in the vegetative period. Once the reproductive period was established, the biosynthesis of the oxygenated monoterpene 1,8-cineole increased [2].

E-Nerolidol is one of the main components of a characteristic scent composition that is colloquially referred to as a ‘white olfactory image,’ a composition that is often varied by diurnal rhythms so as to concentrate the effect at night to attract nocturnal visitors [105,106]. The volatile headspace that is created in the context of plant–insect interactions is often referred to as a ‘scent bouquet’ or ‘volatiles bouquet’. In many species, it has been demonstrated that in response to herbivory, a terpene synthase is activated, which produces E-nerolidol [107,108]. This terpene synthase becomes a rate limiting step in the metabolic product of E-nerolidol, which is then converted by a cytochrome p450 monooxygenase into the homoterpene 4-8-dimethylnona-1,3,5-triene, which allegedly attracts predators of the herbivores [109]. The effect cannot be replicated by mechanical injury to the leaf or by fungal infection, rather, it is a tightly controlled process triggered by specific herbivores. For example, a study with *Cucumis sativus* L. (Cucurbitaceae) demonstrated that attacks by constitutive herbivores lead to the increased expression of E-nerolidol synthase, which cascaded into the accumulation of the intermediate 4,8-trimethylnona-1,3,5-triene [107]. Hence, there is a premise in the literature that herbivore-induced volatile emissions would be facilitated by the ability to alter expression of genes that encode stages in their biosynthesis [110]. This is something that could be investigated in the context of chemical ecology of *P. gaudichaudianum*.

On the other hand, it is also described in the literature that the recognition of the homoterpene emission leads to a reduction in the pollinator’s interest in the species or in the pollen transfer efficiency [111]. Another theory applied to *P. gaudichaudianum* is that the volatiles of leaves and inflorescences act synergistically to attract visitors. Differences in the chemistry of leaves and inflorescences are common, as plants depend on pollinators and attract them to their flowers, yet repel leaf herbivores [112]. In a study with *Nicotiana attenuata Torr. ex S. Watson* and *Datura wrightii Regel* (Solanaceae), it has been demonstrated that the presence of leaf odor further increases the attraction of moth pollinators to the mixture of floral odours and, hence, the flowers. This interaction of mixtures of flowers and leaves can, therefore, be seen as a strategy to optimize the olfactory message and, thus, improve the orientation of the food source based on odors, without risk of the mistaken attraction of herbivores [113]. These chemical synergies between different organs of the same plant specimen have also been alluded to in Citrus [114].

Another point to be highlighted refers to a study that evaluated the variations of Piper ‘herbivoria’ by Eois (specialized moth herbivores) in different forest patterns (dry and wet) and variations in abiotic factors. It was observed that the incidence of parasitism by Eois increased significantly with the increase of precipitation, mainly in humid forests [115]. This leads to the hypothesis that *P. gaudichaudianum*, throughout its evolutionary history, has adapted in order to acquire this chemical phenotypic plasticity (increase of E-nerolidol) as a response mechanism to herbivory by the moth, Eois. The diurnal fluctuations of E-nerolidol observed in the current study may, therefore, have some relationship to selective pressures by herbivory.

### 2.4. Circadian Rythm Variation in the Essential Oil

**Figure 4:**
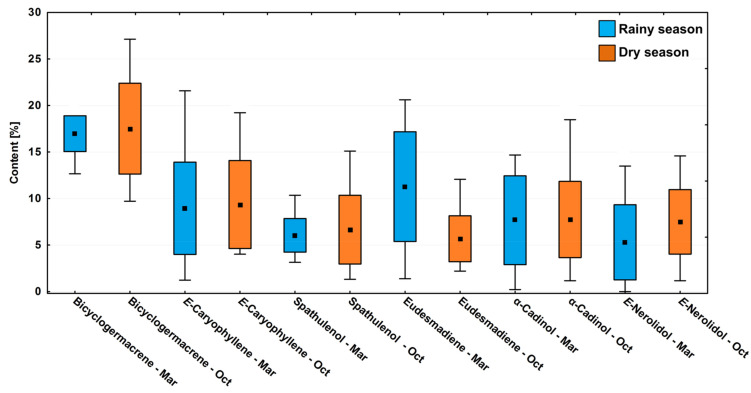
Box plot analyses of the major compounds (%) present in the essential oils from leaves of *Piper gaudichaudianum* Kunth (Piperaceae) in the circadian rhythm study from 12 a.m. to 12 p.m., during Rainy (March) and Dry (October) seasons. Means followed by different letters are significantly different using Tukey test (*p* < 0.05).

In the circadian study, a significant variation (*p* < 0.05) was observed in the contents of the main compounds bicyclogermacrene (R: 13.3–19.7%; D: 10.2–28.6%), E-caryophyllene (R: 1.3–22.7%; D: 4.2–20.2%), eudesmadiene (R: 1.5–21.7%; D: 2.3–12.7%), E-nerolidol (R: 0.3–14.2%; D: 1.2–15.3%), α-cadinol (R: 0.2–15.4%; D: 1.9–19.4%) and spathulenol (R: 3.3–10.9%; D: 1.4–15.9%) (Figure 4). The average relative percentages in the driest period were higher than in the rainy season. However, the effects between the dry and rainy periods on the chemical composition showed no significant difference (*p* > 0.05) (Figure 4).

**Figure 5:**
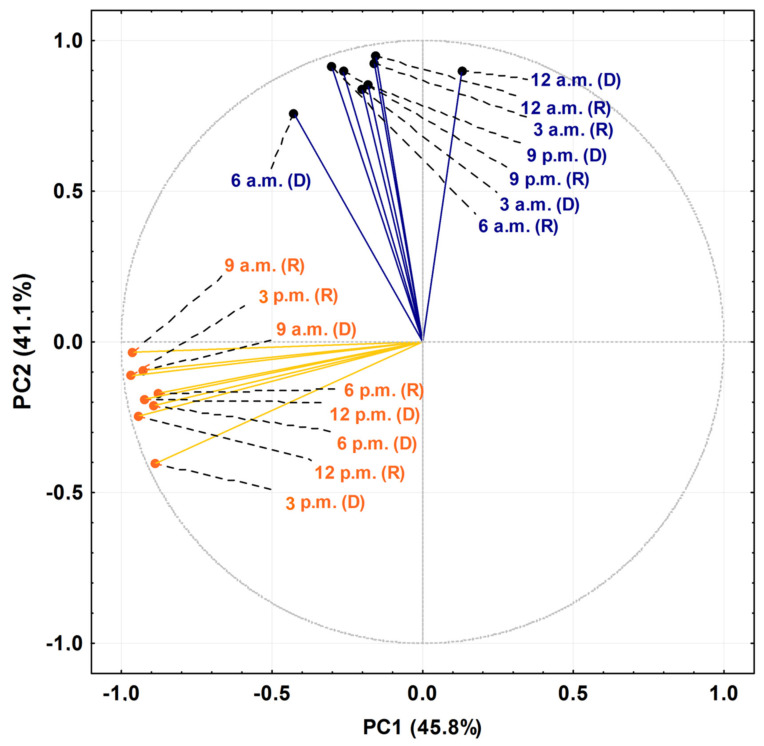
Biplot (Principal Component Analysis—PCA) resulting from the analysis of the essential oils from leaves of *Piper gaudichaudianum* Kunth (Piperaceae) in the circadian study, during the Rainy (R, March) and Dry (D, October) seasons, from 12 a.m. to 12 p.m.

**Figure 6:**
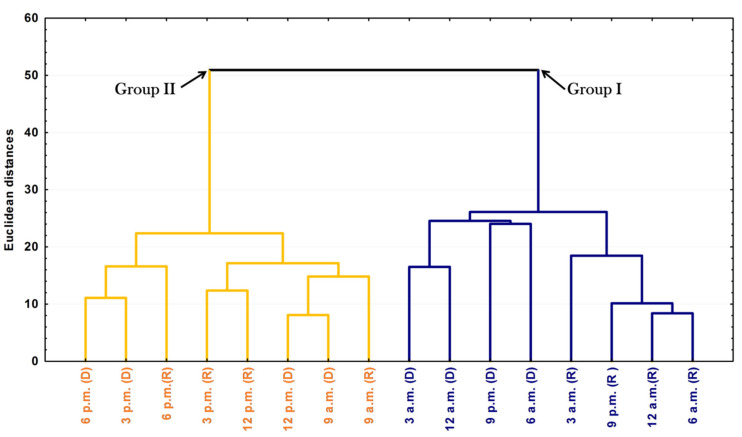
Dendrogram representing the similarity relation of the essential oils from leaves of *Piper gaudichaudianum* Kunht (Piperaceae) in the circadian study, during the Rainy (R, March) and Dry (D, October) seasons, from 12 a.m. to 12 p.m.

The PCA and HCA studies were applied to the chemical profiles from the rainy and dry periods of the circadian study and are presented as Figure 5 and Figure 6. The PCA showed a total variance of 90.8%, and the main components, PC1 and PC2, presented proportional values between themselves, 45.8% and 41.1%, respectively. The two-dimensional axial system generated by the PCA (Figure 5) clearly showed the discrimination of two groups due to chemical variability: Group I—rich in bicyclogermacrene, eudesmadiene, α-cadinol and spathulenol; and Group II—rich in bicyclogermacrene, E-nerolidol and E-caryophyllene. The HCA analysis corroborated the PCA analysis, demonstrating the formation of these two groups (Euclidean distance of 51.0), correlating this difference between the day (9 a.m. to 6 p.m.) and the night (9 a.m. to 6 a.m.) (Figure 6). Analyses of the variation in a smaller Euclidean distance (26.1) showed that at dusk there was a distinction between the rainy (R) and dry (D) periods, increasing the eudesmadiene content in the dry period.

**Figure 7:**
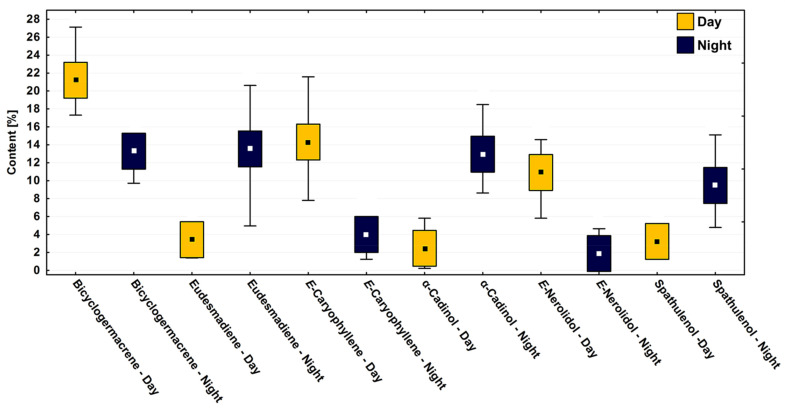
Box plot analyses of the major compounds (%) present in the essential oils from leaves of *Piper gaudichaudianum* Kunth (Piperaceae) in the circadian rhythm study from days (9 a.m. to 6 p.m.) and nights (9 p.m. to 6 a.m.), during March (Mar, rainy season) and October (Oct, dry season). Means followed by different letters are significantly different using Tukey test (*p* < 0,05).

Differences were observed in the variance between day and night (paired ANOVA, F_11.77_ = 25.22, *p* < 0.001) when testing the hypothesis observed in the multivariate analysis. The set of abiotic factors, i.e., temperature, humidity, and radiation, which correlate to the day and night parameters, had more influence on the chemical composition of *P. gaudichaudianum* EOs than the variations between the dry and rainy seasons. The analysis of all the major compounds followed this pattern of day vs. night (Figure 7). For example, in both seasons, the compound bicyclogermacrene was expressed at a constant relative percentage if controlled for timing of collection, demonstrating low variation throughout the seasons (Figure 4) but pronounced differences between day and night (Figure 7). It was noticeable that the period of the day increases the average content (~21%) of bicyclogermacrene (Figure 7). During the day, the compounds E-caryophyllene and E-nerolidol reach their peak and decrease in the night period. Interestingly, E-nerolidol registered its maximum content at 12 p.m. (Table 2; Figure 7). Alternatively, the compounds eudesmadiene, α-cadinol and spathulenol peaked at night, increasing by up to fourfold compared to the day (Table S2—Supplementary Material). The changed patterns of expression between components are seemingly quantifiably reciprocal.

The Pearson’s correlation analysis (Table 3) demonstrated high values of significant direct correlations and inversely proportional to radiation in both periods with the main compounds mentioned above. The oxygenated sesquiterpene E-nerolidol deserves special attention, as it presented an outstanding significant correlation with radiation, temperature, and humidity (*p* < 0.01). In the literature it is reported that most plants emit spikes of volatile terpenoids at noon or in the early afternoon, regulated by light or the internal circadian clock [116]. Allegedly, the increase in radiation elicits genes related to the sesquiterpene biosynthesis. This has been observed in other plant species. For example, the content of E-nerolidol increased according to the UV-B creep rate in young and mature leaves of *Vitis vinifera* L. Vitaceae [117]. This observation reinforces the hypothetical role of E-nerolidol in the protection of *P. gaudichaudianum* against herbivores or parasites. In addition, terpenoids have been recognized for their protective role in high temperature conditions and other environmental stresses [118,119].

Hence, our data provides substantial evidence of a possible chronotype for the essential oil from leaves of *P. gaudichaudianum* [120], where a chronotype is a circadian rhythm ‘type.’ The chronotype is also associated with differences in time between the various physiological events at the different spatiotemporal scales [121,122].

### 2.5. Biosynthetic Considerations

In the seasonal (S) and circadian (C) study, the compounds identified and their respective percentages in the *P. gaudichaudianum* EOs were grouped according to their respective carbon skeletons [80,123,124]. The results are shown in Table 4 and Table 5, respectively. It was possible to find a total of 19 carbon skeletons (S: 19; C: 15), being 4 for monoterpenes and 15 for sesquiterpenes. The five main carbon skeletons (C-skeletons) were bicyclogermacrane (S: 11.2–23.2%; C: 10.2–28.6%) > aromadendrane (S: 2.1–19.3%; C: 5.0–19.3%) > eudesmane (S: 5.8–16.2%; C: 2.3–25.4%) > cadinane (S: 2.2–14.6%; C: 1.7–27.8%) and > farnesane (S: 3.3–22.9%; C: 0.0–16.1%). The C-skeletons with greater diversification (greater number of compounds) were cadinane (S: 22; C: 18) > eudesmane (S: 12; C: 9) > aromadendrane (S: 8; C: 6) > caryophyllane (S: 8; C: 4).

Comparing the percentages between C-skeletons, it was found that the contents of compounds with bicyclogermacrane were in high percentages during the year, suggesting that the central precursor germacrane has its production favored during this period. The increase in aromadandrane is linked to the decrease in germacrane (r= −0.685; *p* = 0.02), suggesting that the production of compounds with aromadandrane C-skeletons, whose precursor is bicyclogermacrane, is conditioned to displacement due to consumption, almost total, of substrates with a germacrane skeleton. Specifically, the bicyclogermacrene expression was reduced in August and September when cadinane expression was favored. However, bicyclogermacrene synthesis displaces cadinane synthesis by up to two times. The percentages of caryophyllane derivatives were low compared to the other sesquitepene C-skeletons. The increase in the humulane skeleton is proportional to the increase in the caryophyllane skeleton, plainly by the fact that these two compounds share a common biosynthetic route. Compounds with elemane skeletons were favored in the months of May to August (intermediate rain rates) (Schemes S1 and S2).

In the circadian study, basically the same pattern of diurnal expression, between day and night, was observed in both seasons (Table 5, Scheme S3). To an extent, some of this variation could also be correlated to abiotic factors. In other studies, the changes in metabolite expression are also correlated to short-term changes to the expression of the genes associated with the Metileritritol-phosphate (MEP) and Mevalonate (MEV) pathways [116,125,126]. It is commonly observed that the expression of secondary metabolites can be correlated to abiotic factors, such temperature, humidity, intensity and quality of light [116,125,126,127].

In the dry and rainy seasons, the chemical profiles were characterised by 12 and 15 C-skeletons, respectively. Monoterpenes were present in trace quantities in the rainy season. Expression of monoterpenes was increased during the day. A similar scenario is seen with the sesquiterpenes, which were higher from 9 a.m. to 3 p.m. Furthermore, the ratios of sesquiterpene classes change in that period, reinforcing the hypothesis that the biosynthesis changes. The notion that the species changes biosynthesis during the peak light period reiterates that genes regulate the expenditure of resources to maximise on efficiency.

It is interesting that bicyclogermacrene synthesis during the day is reciprocal to spathulenol synthesis during the night. It is well known that spathulenol is an artefact of bicyclogermacrene oxidation in several species [128,129]. Bicyclogermacrene often converts into spathulenol during hydrodistillation or spontaneously in the essential oil as it ages. It may be feasible, in this case, that bicyclogermacrene biosynthesis is inactive during the night period, at which time the accumulated bicyclogermacrene is slowly converted into spathulenol. This possibility should be considered in the case of other components that increase during the night period (from 9 p.m. to 6 a.m.), such as the cadinane and eudesmane derivatives. This may explain the biosynthetic contradiction that is apparent, involving the contrasting synthesis of acyclic and monocyclic C-skeletons, in day, which requires lower energy costs for construction and structural specialization for production. This contrasts to the night period favoring the production of bicyclic and tricyclic C-skeletons, which demand greater expenditure on energy in construction and structural specialization for production.

Nevertheless, the biological properties of this plant are evidently related to the collection time. From an ecological point of view, this sheds light on the time-window to obtain EOs used in the construction of experimental models of baits for bats [3,15,16,18]. So far, it is known that the emission of volatile compounds is important for these animals [16]. The specific roles of the individual components require clarification, but as a whole, the volatile bouquet is comprised of allelochemicals (cairomonas, alomonas or sinomonas) that trigger an olfactory response in bats.

### 2.6. Reduction-Oxidation Indices

In circadian and seasonal studies of EOs from *P. gaudichaudianum*, the compounds were analyzed in relation to their oxidation number (N_OX_), following the Hendrickson–Cram–Hammond [77] rules on the sum of the oxidation states of each atom of the molecule and oxidative steps (OS) [79]. As mentioned by Sayuri et al. [80], N_OX_ does not allow the comparison between different chemical classes, since the number of carbon atoms between chemical classes is usually different. These authors proposed the OS calculation, which is obtained by subtracting the N_OX_ of the compounds of interest from the N_OX_ of the common biosynthetic precursor of that C-skeleton, a result divided by two according to Equation (1) [79]:

(1) $$\mathrm{OS}=\frac{\left(N_{ox}{}_{\mathrm{precursor}}-N_{ox}{}_{\mathrm{Compound}}\right)}{2}$$

The EO compounds from *P. gaudichaudianum* showed N_OX_ ranging from −10 to −38 (Figure S1—Supplementary Material). The monoterpenes´ N_OX_ were between −16 and −10. The bicyclic monoterpene camphor showed the highest N_OX_ of -10. For sesquiterpenes, the N_OX_ values were determined as being between −24 and −16. The highest N_OX_ was for cadalene (−16). However, most terpene compounds with higher N_OX_ values are found in the EO at much lower relative percentages.

In this static analysis model based on N_OX_ and OS, for the two studies (seasonal and circadian), when comparing the values with the precursor of the respective chemical class or terpene type, it was observed that the generated compounds kept the numbers of OS (0 to 2) constant (Figure S1—Supplementary Material). Hence, these values provided very limited detail and were not useful in characterising phenetic patterns. In most cases, the increased metabolite diversity was not recognised by these values, as the oxidative steps value did not change relative to its precursor. This was recognised in an earlier study in Asteraceae and in the analysis of temporal patterns of skeletal production in essential oils of *Baccharis microdonta* Steud. ex Baker and *B. elaeagnoides* DC. [80].

Furthermore, the redox theory developed in the early 1990s by Gottlieb [81] argued that the evolution of oxidative pathways in plants occurred as a protective mechanism against oxidative damage, reflected directly in the role of atmospheric oxygen, which increased during the time of life on earth. Although this theory is outdated and disproven, the redox indices developed by these earlier chemical ecologists continue to show patterns, although the modern scientific approach is to acknowledge that such patterns are no longer considered validating to an old theory.

Nevertheless, the continued realization of chemical patterns in oxidative metabolites has led to the development and application of static quantitative methodologies (chemosystematic) to assess the phenotypic responses to taxa to abiotic factors. It is well known that redox reactions are part of the metabolic cycle of plants, mainly in the physiological processes of plastic responses to seasonal variations and under the command of the circadian clock [130]. This process normally involves complex chemical mechanisms. Assessing these molecular oxidation patterns of a mixture is necessary to really understand the redox mechanism on a fluid time scale and based on a non-static model. Hence, the OS value is not considered useful in the context of the current study.

Here, we propose a new intermediate equation named the Weighted Average Redox Standard (S_RO_) given by the oxidation number of the compound of interest (N_OX_) multiplied by the quantification value obtained in the analyzed sample (Q%) and divided by the number of carbon atoms in the molecular skeleton (n), according to Equation (2):

(2) $$S_{RO}=\frac{N_{ox}{}_{\mathrm{compaud}}\times Q\%\;}{n}$$

Equation (2) gives a weighted average value of the oxidation of the carbon atoms of the compounds (S_RO_). This equation is considered an intermediate step to obtain the General Mixture Redox Index (GM_RO_, or Ramos and Moreira´s index for mixtures). The calculation is provided as Equation (3). This index helps to understand a complex mixture of compounds. To do this, it is necessary to calculate the sum of the S_OR_ of all compounds divided by the number of compounds identified in the sample (N_CI_), according to the new Equation (3):

(3) $${\mathrm{GM}}_{RO}=\frac{\sum S_{RO}\;}{N_{CI}}\;$$

When this new equation is applied to chemical ecology studies, we propose to name it the Ecological Mixture Redox Index (EM_RO_).

Attention should be given to the need for standardization of the applied quantification technique (for example, GC-MS, GC-FID, HPLC-MS and UPLC-MS) to allow data comparison and to guarantee the best quantification to reduce the interfering results. The calculation fails if comparison of the EM_RO_ is made between samples that were quantified using different methodologies. This index is explained by the higher the value obtained (closer to zero), the greater the oxidation of the compounds in the mixture and visa-versa; the more distant a value is from zero, the greater the reduction is.

This index (GM_RO_ or EM_RO_), conceptually, can be applied broadly in ecology, pure chemistry and product development (authentication). For example, in chemical ecology, at the level of α-chemodiversity, it can predict and explain a pattern about changes in metabolism induced by abiotic and ontogenic factors, as well as interactions in ecological niches. At the level of β-chemodiversity EM_RO_ can correlate and explain phenomena related to the adaptive fluctuations of the special metabolism of specimens in different sites. Finally, at the level of γ-chemodiversity, this index helps to understand changes at the interspecies level occurring through ecological succession [59].

The results of the GM_RO_ or EM_RO_ calculations from the seasonal and circadian studies of EOs of *P. gaudichaudianum* are presented in Table 1 and Table 2. Variation was evident from -6.4 to -3.6 in the diurnal study (S: −5.6 to −3,6; C: −6.4 to −3.6). In the annual variation (January to December 2017), the reproductive period (January, February, November and December) coincided with the highest values of GM_RO_ (greatest oxidation). After periods with high rainfall, the GM_RO_ values showed a decrease (greater reduction). In the same period, there were decreases in the diversity of substances present in the EOs. However, the results of Pearson’s correlation for the annual variation did not show significant values.

**Figure 8:**
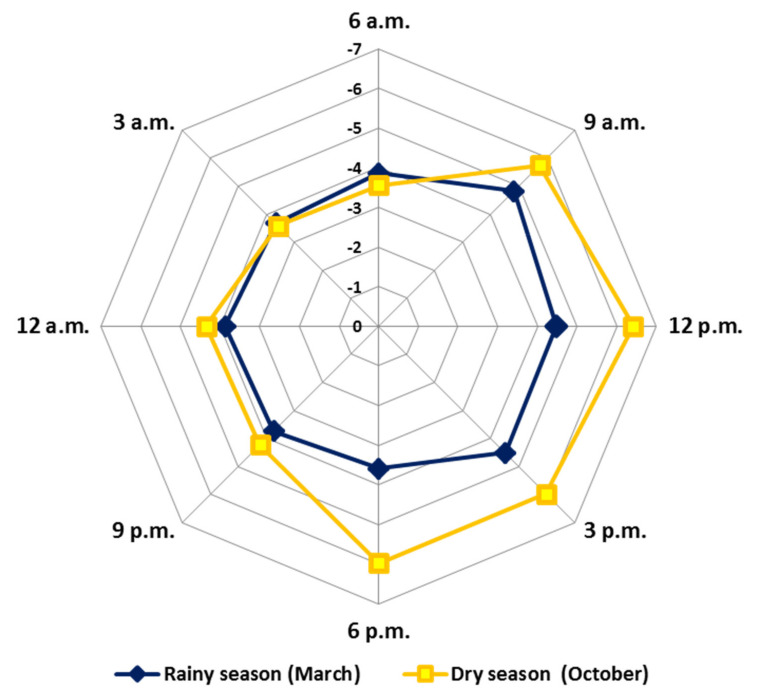
Radar plot representation of the General Mixture Redox Index obtained from essential oils from leaves of *Piper gaudichaudianum* Kunth (Piperaceae) in the circadian rhythm study from 12 a.m. to 12 p.m., during March (Mar, rainy season) and October (Oct, dry season).

Figure 8 (radar graph) shows the GM_RO_ variation values obtained for the EOs of the circadian study. The results demonstrated that the average values showed a significant difference during the days (October and March) and between the periods (*p* = 0.05). The average in the rainy season (March) (R: −4.8 to −3.6) was higher (more oxidised) than the dry season (October) (D: −6.4 to −3.6). The mixtures of the compounds present in the EOs showed greater reduction over the days (R: −4.8 to −3.7; D: −6.4 to −4.2) and greater oxidation at night (R: −3.9 to −3.7; D: −4.3 to −3.6). Pearson’s correlation showed significant correlations between relative humidity, precipitation and radiation. These results describe the natural metabolic movement that leads to a possible redox balance throughout the day. In addition, our GM_RO_ results reinforce the protection hypothesis that specialized metabolites exert under stress conditions to minimize the formation of reactive oxygen species (ROS) and reactive nitrogen species (RNS). All these variations aim to guarantee the full functioning and maintenance of plant physiology [130].

A study comparing the effects of adaptation and damage to vine leaves showed that the metabolism of isoprenoids was modulated according to UV-B rates. In addition, this study associated the damages caused to the generation of ROS with the increase in the excitation energy [117]. In the literature, it is described that volatile terpenoids (monoterpenes and sesquiterpenes) are quickly combined with ROS and that these reactions are stimulated by changes in light and temperature conditions [117,131]. Likewise, the data obtained from GM_RO_ at the macro-metabolic level corroborate the redox theory with a quantitative parameter, which postulates that, at the oxidation level, the specialized metabolism requires the existence of binary antioxidant systems: meaning that there will be a balance to guarantee a proportion of different classes of compounds in the redox system. Therefore, compounds may vary in quantity (abundance) or in reducing power (high potential), to achieve “general reducing power”, considered a metabolic homeostasis [81].

**Figure 9:**
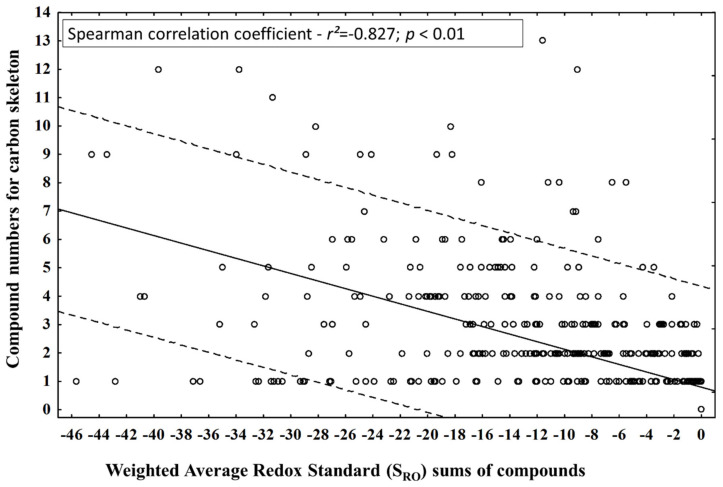
Correlation between compound numbers for carbon skeleton and Weighted Average Redox Standard (S_RO_) for the compounds identified in the essential oils from leaves of *Piper gaudichaudianum* Kunth (Piperaceae) in the seasonality and circadian rhythm studies.

This theoretical statement led to the question: does the diversification of the number of compounds by carbon skeletons during different periods (seasonal and circadian study) lead to an increase in oxidation or a reduction in the compounds of the EOs from leaves of P. gaudichaudianum? The number of compounds in each carbon skeleton and the S_RO_ values obtained in seasonal and circadian studies did not have normal distributions (Kolmogorov-Smimov test), so Spearman’s nonparametric test was applied to correlate them (Figure 9) and to answer the question. It was possible to observe a significant inversely proportional correlation between the tested parameters, suggesting that the diversification of the carbon skeleton is followed by an increase in the S_RO_ of the compounds.

However, the scatterplot (Figure 9) clearly shows that the diversification and the increase in the reduction do not occur homogeneously. This fact is related to issues of biosynthetic route. For example, bicyclogermacrane has only one member compound, and the weighted quantitative variations lead to the most reducing state in the mixtures by decreasing the S_RO_. In fact, this compound is a biogenic intermediate for the formation of compounds with aromadendrane-based skeletons. Consequently, the latter showed a higher pattern of oxidative and structural diversification than bicyclogermacrane in a biogenic compensatory way. The same fact was observed for germacrane in relation to cadinane and eudesmane. Based on Gottlieb’s redox theory, it is possible to formulate the hypothesis that the diversification of carbon skeletons in the biosynthetic routes leads to an increase in the level of weighted average oxidation (S_RO_) as a biosynthetic control. However, the quantitative percentage increases in the main intermediate metabolites (precursors) are responsible for guaranteeing the retraction of skeletal diversifications and, consequently, of the generalized oxidation of *P. gaudichaudianum* EO compounds. This evidence demonstrates that static models, such as OS analysis, do not clearly reflect biosynthetic movements at different time scales, so we suggest the new S_RO_ and GM_RO_ indices. Thus, we make a statement of PoC related to the two new indices (S_RO_ and GM_RO_) that can be applied to study the redox of complex mixtures.

### 2.7. Chemophenetic Aspects in Piper Gaudichaudianum

**Figure 10:**
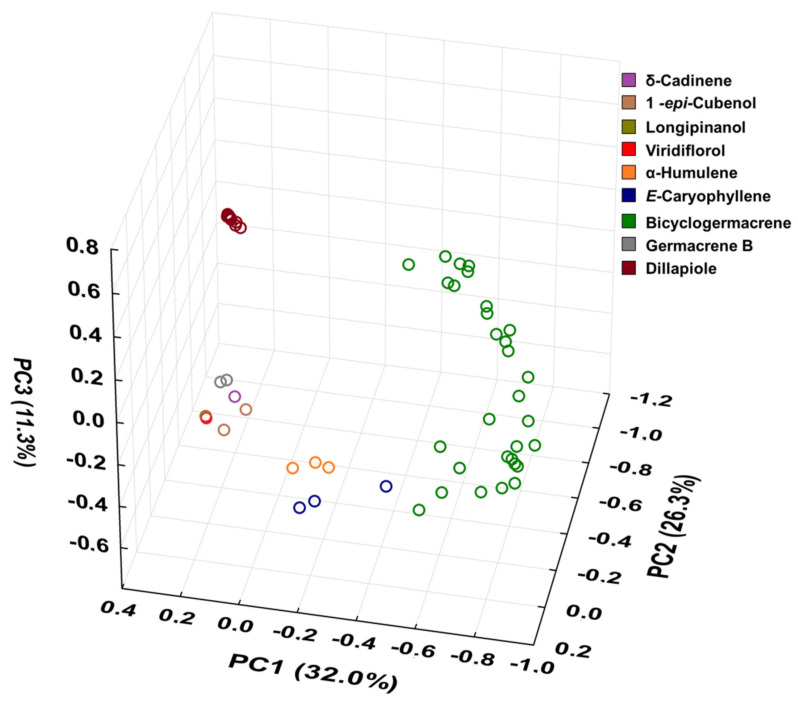
Triplot (Principal Component Analysis—PCA) resulting from the analysis of the 60 essential oils composition from leaves of *Piper gaudichaudianum* Kunth obtained in this study (seasonality study—RJ1-12; circadian study—RJ13-29) and from literature database RO [41]; RS1-1 to 16 [43]; RS2 [38,45]; RS3 [42]; RS4 [44]; SC [39]; SP1-1 and 2 [38]; PR1 [40]; PR2 [47]; PR3-1 and 2 [93]; PR4 [94]; PR5-1 and 2 [95]; PR6 [46]; and PR7 [46].

**Figure 11:**
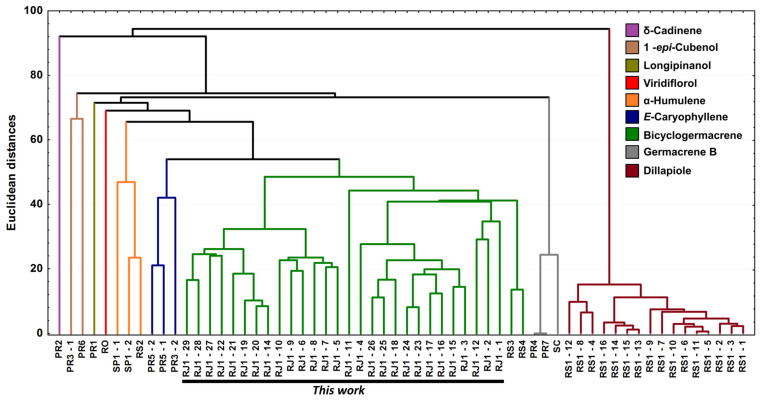
Dendrogram representing the similarity relation of the 60 essential oils composition from leaves of *Piper gaudichaudianum* Kunth obtained in this study (seasonality study—RJ1-12; circadian study—RJ13-29) and from literature database RO [41]; RS1-1 to 16 [43]; RS2 [38,45]; RS3 [42]; RS4 [44]; SC [39]; SP1-1 and 2 [38]; PR1 [40] PR2 [47]; PR3-1 and 2 [93]; PR4 [94]; PR5-1 and 2 [95]; PR6 [46]; and PR7 [46].

Based on data from this study and those from the literature, 60 (n = 60) EOs have been characterised chemically (Table S3—Supplementary Material) from *P. gaudichaudianum* leaves. The data were processed and analyzed by PCA and HCA and are shown in Figure 10 and Figure 11, respectively.

For better results considering the data set, the PCA was built in three axes, with a total variance of 69.6%, being PC1 (32.0%), PC2 (26.3%) and PC3 (11.3%). From these data it was possible to observe the initial separation of two groups (Figure 10): Group I—with less variability, with a predominance of the shikimate pathway and with positive charge on the PC1 and PC3 axis and negative on PC2 (Dillapiole); and Group II—with greater variability, with a predominance of comounds that follow the acetate-mevalonate (MEV) and metileritritol-phosphate (MEP) biosynthetic routes and with loads distributed in opposition to the previous group (α-humulene, E-caryophyllene, δ-cadinene, 1-epi-cubenol, longipinanol, viridiflorol, germacrene B and bicyclogermacrene). The compounds that most contributed to the separation of the groups in PC1 and PC2 with negative charge were bicyclogermacrene (−20.3) and dillapiole (−23.4), respectively. PC3 was responsible for the smallest variations between samples. The values found were remarkably close to each other. HCA (Figure 11) showed higher rates of similarity but confirmed the separation of those groups assigned in the PCA.

From the combined analysis of PCA and HCA, it was possible to define nine (n = 9) possible different chemotypes for *P. gaudichaudiaum*: chemotype δ-cadinene, with one sample (PR2) [47]; chemotype 1-epi-cubenol, with two samples (PR3-1; PR6) [46,93]; chemotype longipinanol, with 1 sample (PR2) [40]; chemotype viridiflorol, with 1 sample (RO) [41]; chemotype α-humulene, with 3 samples (SP1-1 to 2 and RS2) [38,45]; chemotype E-caryophyllene, with 3 samples (PR3-2 and PR5-1 to 2) [93,95]; chemotype germacrene B, with 3 samples (PR4, PR7 and SC) [39,94,100]; chemotype dillapiole, with 16 samples (RS1-1 to 16) [43]; and chemotype bicyclogermacrene, with 30 samples (RS3, RS4 and this study—RJ1 1 to 28). The RS3 and RS4 samples showed high percentages of E-nerolidol (22.6–24.4%) and α-humulene (21.3–21.3%) and a lower percentage of bicyclogermacrene (7.4–13.2%).

The percentage content of this last compound led to the grouping of RS3 and RS4 on chemotype bicyclogermacrene. Although we cannot rule out negative biases around plant collection errors (schedules, season), transportation, identification, quantification or the detection of compounds, they are unlikely to significantly affect the dillapiole, bicyclogermacrene, germacrene B and E-caryophyllene clusters identified by different research groups and different specimens. These results clearly show the plastic chemical response capacity observed by *P. gaudichaudianum* EO to edaphoclimatic and biotic factors.

**Figure 12:**
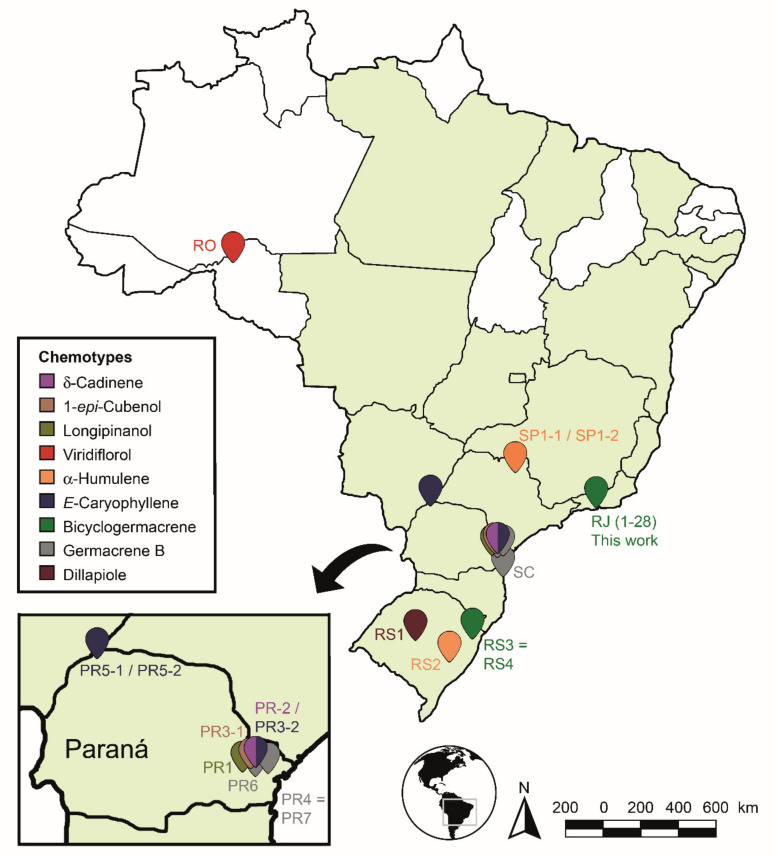
Spatial distribution of *Piper gaudichaudianum* Kunth chemotypes in Brazil in accordance with this study (seasonality study—RJ1 to 12; circadian study—RJ13 to 29) and from literature database RO [41]; RS1-1 to 16 [43]; RS2 [38,45]; RS3 [42]; RS4 [44]; SC [39]; SP1-1 and 2 [38]; PR1 [40] PR2 [47]; PR3-1 and 2 [93]; PR4 [94]; PR5-1 and 2 [95]; PR6 [46]; and PR7 [46].

The identified chemotypes were distributed on the Brazilian map to demonstrate the chemogeographic distribution of the species (Figure 12). The species areas of occurrence were highlighted in green according to the Flora of Brasil 2020 Project [132]. It was observed that the samples found and analyzed were grouped mainly in the South and Southern regions of Brazil (Rio de Janeiro, São Paulo, Paraná and Rio Grande do Sul States), except for a sample collected in Rondônia State (North region). The samples showed high levels of non-oxygenated sesquiterpenes (8.3–81.5%) in relation to the other compounds. Only a few samples in the State of Paraná showed significant amounts (7.1–22.8%) of monoterpenes.

We highlight, considering the analyzed data set, that the production of monoterpenes is not favoured in *P. gaudichaudianum* EOs, but it does favour sesquiterpenes. Some samples showed higher amounts of compounds from the shikimate pathway in Southern Brazil: dillapiole (70.5–57.8%—RS1-1 to 16) and myristicin (15.2%—PR6). So, we emphasize more in-depth evaluations at different time scales for all samples in the State of Paraná, since this great chemical plasticity may suggest not only a chemotypic variation, but a possible formation of geotypes. This region has a concentrated level of chemical plasticity relative to the other sites sampled. However, the PCA and HCA data set used in this study to recognise the chemotypes has demonstrated similar surprising outcomes in the literature for other plant species [62,133,134].

The results for the EOs from *P. gaudichaudianum* registered in the literature 31 (n = 31) types of carbon skeletons (Scheme S4 and Table S3—Supplementary Material). One (n = 1) C_6-_C_3_ derivative (miristicin, eugenol and dillapiole) (alkylbenzene); one (n = 1) derived from C_6_-C_6_ benzoic acid (benzyl benzoate); one (n = 1) chromene (eupatoriochromene); and twenty-eight (n = 28) from the MEP and MEV pathways, mainly farnesyl pyrophosphate products.

The C-skeletons generated via the MEP and MEV pathways with the highest qualitative occurrence (presence and absence) were caryophyllane (n = 58), aromadendrane (n = 56), humulane (n = 56) and germacrane (n = 55). The propensity for routes in the production of compounds with humulane and caryophyllane C-skeletons is found qualitatively and quantitatively in the samples (n = 59). An exception is evident for PR1, in which the production of the longipinane C-skeleton was favored, a tricyclic compound structurally more complex than humulane, that is, the precursor to longipinane.

Correlating the relative percentage of the compounds by the C-skeleton and the latitude (Lt) and longitude (Lg) (data from literature and from our study), it was observed that there was a significant (*p* < 0.05) and directly proportional increase in geographic position with the quantitative percentage of the compounds germacrane (Lt: r = 0.563; Lg: r = 0.578), bicyclogermacrene (Lt: r = 0.572; Lg: r = 0.793) and aromadandrane (Lt: r = 0.532; Lg: r = 0.508). So, these data suggest a longitudinal and latitudinal quantitative biosynthetic gradient from the Tropic of Capricorn to the Equator for the formation of compounds with germacrane carbon skeletons towards aromadandrane. It was also found that the formation of possible chemotypes showed greater chemical structural (skeleton) diversification and did not present spatial homogeneity in the distribution of chemical phenotypes (chemical compound) in relation to their logitudinal and latitudinal occurrence. Most chemotypes showed diversification in skeletons centered on biogenetic derivatives or compounds with a germacrane or humulane skeleton, following the biosynthetic path of germacrane (PR4; PR7 and SC); cadinane (PR2) and cubebane (PR6 and PR3-1) or germacrane; and bicyclogermacrane (RJ1 to RJ28, RS3 and RS4) and aromadandrane (RO). However, when the precursor was humulane (RS2, SP), it followed the biosynthetic pathway for the formation of caryophyllane (PR3-1) or longipinane (PR1).

Thyme (*Thymus vulgaris* L.), a pioneer and invasive species in several countries, showed phenotypic chemical modulations in the terpenes present in the EOs in different geographical positions and under evaluation in the edge effect. It was reported that the chemical response of plasticity was mainly related to environmental factors and that the most important mechanism for successful plant invasion at the forest edge is associated with the presence of the carvacrol type chemotype [134]. This is in favor of the argument of the structural (skeleton) spatial diversification of the chemotypes present in *P. gaudichaudianum*, which also has pioneering characteristics.

## 3. Materials and Methods

### 3.1. Plant Material and Experimental Design

Leaves from *Piper gaudichaudianum* Kunth were collected in the Atlantic Forest, in the Tijuca National Park region, Rio de Janeiro—RJ, Brazil (22°58’13” S, 43°14’34” W, Elevation: 452 m) from January to December 2017. Authorization for the collection of botanical material was given by the Chico Mendes Institute for Biodiversity Conservation (ICMBio), number 57296–1. Samples of the fertile specimens were collected, identified and deposited with voucher number RB730964 at the Herbarium of the Botanical Garden of Rio de Janeiro (JBRJ), Rio de Janeiro, Brazil. This study was registered with the Genetic Heritage Management Council under identification AE20045. The experimental design consisted of 12 collections of leaves from specimens for the seasonality study and 16 collections for the circadian rhythm study. For the seasonal study, 100 g of leaves were sampled monthly on the 15th day, at 9 a.m., from January to December 2017. For the study of circadian rhythm, samples were obtained from the same specimen every three hours, with collections performed at 12 p.m., 3 p.m., 6 p.m., 9 p.m., 12 a.m., 3 a.m., 6 a.m. and 9 a.m. on 14 March and 15 October 2017. These two sequences of collections correlate to the rainy and the dry seasons, respectively. Data on abiotic factors, including average temperature (°C), precipitation (mm), radiation (KJm^−2^) and humidity (%) of the collection site were obtained from the Brazilian Institute of Metrology and Research (INMET) for the weather station (A652-OMM: 86887) and are shown in the Supplementary Materials Figure S2.

### 3.2. Essential Oils Production and Analysis

The collected leaves were manually crushed and subjected to hydrodistillation for two hours in a Clevenger-type apparatus. The EOs were dried over anhydrous sodium sulphate (Na_2_SO_4_, Sigma-Aldrich, Brasil) and the total EO yield was expressed as the percentage value related to fresh plant material (g/100 g) [2,96,135].

EOs were diluted in dichloromethane (1 mg/mL) (Tedia, Brazil) and submitted to analyses by Gas Chromatography coupled to Mass Spectrometry (GC-MS) to assist in the identification and GC coupled to a flame ionization detector (GC-FID) to compound quantification.

GC-MS analyzes were performed using the HP—Agilent 6890N gas chromatograph equipped with an automatic GC sampler with 120 positions and coupled to a model 5973 (MS) mass spectrometer. The (5%-phenyl)-methylpolysiloxane capillary column (HP-5MS, 30 m × 0.25 mm I.D., 0.25 μm film thickness) (Agilent J & W; GC columns, USA) was used for all analyses. GC-MS conditions were injector temperature of 270 °C; injection at 1 μL of the EO solution splitless; oven temperature programming from 60–240 °C (3 °C/min); Helium as carrier gas (>99.99%), adjusted at a linear speed of 36.5 cm/s (1.0 mL/min); ionization by electron impact at 70 eV; ionization source and transfer line temperature of 200 and 250 °C, respectively. Mass spectra were obtained by automatic scanning every 0.3 s, with mass fragments in the range of 40 to 600 m/z.

Quantification of volatile constituents was obtained by normalizing the peak area with no correction and using an HP-Agilent 6890 GC Series device, coupled to the FID detector, operated under conditions similar to the GC-MS. The retention index (RI) was determined from the retention time of a homologous series of n-alkanes (C8-C20, Sigma-Aldrich) obtained by GC-FID, under the same conditions of EO analysis. The compounds present in the volatile mixture were identified by comparing the fragmentation patterns of the mass spectra with database records (WILEY 7n, NIST) and comparing the calculated RI [136] with those from literature [137]. In addition, co-injection with authentic standard was done wherever possible.

### 3.3. Statistical and Chemophenetic Analysis

For the analysis of circadian, seasonal and chemophenetic variations, the correlation coefficients between climatic and geographic parameters were calculated by yield, chemical classes, the main constituents and their carbon skeletons. For correlation analysis, using the Kolmogorov–Smimov test, the data set with normal distribution was performed by Pearson’s analysis, and for those without normal distribution, the Spearman analysis was used. Statistical significance was assessed using the Tukey test (ANOVA by Tukey HSD post hoc test). The oxidation state was calculated by using the number oxidation (N_OX_) and oxidative steps (OS) calculations as used by other authors [79]. In addition to performing the proof of concept in the developed indices of Weighted Average Redox Standard (S_RO_) and General Mixture Redox Index (GM_RO_, or Ramos and Moreira’s index for mixtures). Principal Component Analysis (PCA) and Hierarchical Cluster Analysis (HCA) were applied to verify the interrelationship in the composition of leaf EO collected at different time and months. For chemotype analysis, information about the chemical composition of EO published in the literature for *P. gaudichaudianum* was mined. Only chemical data produced by us is included in the current study. These data and mined data were applied to the PCA and HCA matrix to determine the chemotypes [81,138]. The results were processed by STATISTICA version 10 (StartSoft Inc., Tulsa, OK, USA).

## 4. Conclusions

*Piper gaudichaudianum* Eos’ content as well as the relative percentage of the compounds are influenced by the circadian rhythm and season. The highest yield was achieved in the months of December to February, at 6 a.m. in the rainy season and at 12 p.m. in the dry season. The major identified compound was bicyclogermacrene, with variations of *E*-caryophyllene, eudesmadiene, *E*-nerolidol, α-cadinol and spathulenol. We report for the first time the high chemical phenotype plasticity presented by *P. gaudichaudianum* in different time scales. It was possible to correlate changes in chemical composition at different phenological stages and under different abiotic factors. The variation between the dry and rainy periods did not strongly influence the chemical composition, however, there were significant variations in the volatiles between day and night. More complex terpenes (bicyclic and tricyclic) were biosynthetized during the nighttime. That said, a possible chronotype based on the chemical composition of EOs is described for the first time in the genus *Piper*. We demonstrated that C-skeleton types are an important tool for chemophenetic analyses, and their percentage of occurrence showed trends of significant variation in the biosynthetic routes throughout the seasonal and circadian rhythm. Static models of chemosystematic analysis (considering oxidative steps) are not enough to determine oxidation patterns during temporal variations of terpenoids. Thus, for the first time and using *P. gaudichaudianum* as a model and considering the compound quantification of its EO, it was possible to develop and make a proof of concept of a new approach based on the “Weighted Average Redox Standard” (S_RO_) and the “General Mixture Redox Index” (GM_RO_). These calculations led to correlating the production of EO compounds to the general metabolism of the species, demonstrating that there is a direction for a possible redox balance throughout the 24 h of the day. It was also possible to demonstrate that the diversification in the number of compounds per carbon skeleton in the EO of *P. gaudichaudianum* is correlated to an increase in the S_RO_ of the compounds. These oxidative diversifications have as their main control point the quantitative increase in biogenetic precursors. In addition, the chemophenetic approach of *P. gaudichaudianum* allowed us to determine nine possible chemotypes by mining the literature. Considering carbon skeletons, it was demonstrated that most chemotype diversifications are centered on biogenetic derivatives or compounds with a germacrane or humulane skeleton. Despite the diversification of the skeletons of the chemotypes, the data analysis did not corroborate the existence of homogeneous spatial occurrence in the compounds expressed by the chemical phenotypes in a gradient with latitude and longitude. All data together provide evidence of ecological, chemosystematic and chemophenetic significance for the management and conservation of this medicinal and ritualistic species used by the Brazilian population.

## Figures and Tables

**Table 1:** Results of the seasonal analysis of the essential oils obtained from leaves of *Piper gaudichaudianum* Kunth (Piperaceae) collected from January to December 2017. Yields and General Mixture Redox Index (GM_RO_) are also presented. Quantities are averaged out of three replicates.

C-Skeleton	Compounds ^a^	RI_calc_	RI_lit_	Relative Peak Area (%) ± SD											
				Jan	Feb	Mar	Apr	May	Jun	Jul	Aug	Sept	Oct	Nov	Dec
Hexane	3*E*-Hexenol	844	844				tr								
Pinane	α-Pinene	931	932		0.2	0.2	0.3								
Camphane	Camphene	956	954												
Pinane	β-Pinene	975	979	0.6	0.5	0.5	0.1								
Myrcane	Myrcene	985	988				0.3								
Menthane	Limonene	1022	1024												
Myrcane	*Z-*Linalool oxide	1064	1067	0.8			1.2								
Myrcane	Linalool	1093	1095	5.4		1.2	4.3	1.2							
-	Undefined *m/z* 154	1095	-	0.4											
Nonane	*n*-Nonanal	1100	1100		0.1										
Menthane	1-Terpineol	1132	1130	0.3											
Camphane	Camphor	1142	1141	0.1	0.1	4.6	3.5	4.8							
Camphane	Camphene hydrate	1144	1145			0.4	1.4	0.3							tr
Menthane	α-Terpineol	1182	1186	0.2		1.2	6.3	2.1	tr						tr
Camphane	Borneol	1162	1165												
Camphane	Bornyl acetate	1282	1285			tr	0.3								
Undecane	Undecanal	1303	1305								tr				
Elemane	Bicycloelemene	1322	1329	0.7	tr			0.5	0.3	2.3	2.9	3.4	tr		
Elemane	δ-Elemene	1332	1335		0.5	3.2	3.2	5.7	4.6	3.5	6.5			2.6	3.4
Cubebane	α-Cubebene	1345	1348		0.3	tr		1.2			0.4	5.6	3.4	1.2	2.8
Myrcane	Neryl acetate	1356	1359					1.3						0.3	
Copaane	α-Ylangene	1372	1373		0.3										
Copaane	α-Copaene	1375	1374		0.4	1.6		3.8		6.3	5.3	4.9	5.9		3.2
-	undefined *m/z* 202	1376	-	1.2											
Myrcane	Geranyl acetate	1376	1379					0.3	tr	0.4					
Bourbonane	β-Bourbonene	1386	1387		0.1	0.1	tr					0.1			
-	undefined *m/z* 206	1387	-		0.3			1.2						2.3	
Elemane	β-Elemene	1388	1389	1.7	0.7	4.6	2.3	2.3	5.7	4.5	1.9	2.0	3.2	2.6	3.4
Aromadendrane	α-Gurjunene	1409	1409		0.2					2.3	1.1	3.2	4.1		4.6
Caryophyllane	*iso*-Caryophyllene	1411	1409					tr	0.3	1.2	tr				
Caryophyllane	***E*-Caryophyllene**	1417	1419	**3.3**	**8.7**	**9.0**	**6.9**	**7.6**	**10.2**	**11.2**	**5.4**	**7.3**	**9.3**	**4.8**	**3.1**
Copaane	β-Copaene	1428	1430		1.2			1.9		3.2	3.2	2.3	2.4	2.7	2.1
Aromadendrane	β-Gurjunene	1431	1434		0.9						1.2			2.0	2.2
Humulane	β-Humulene	1433	1436							2.3					
Elemane	γ-Elemene	1436	1437	0.8	0.2	1.2		1.8			1.1			1.2	2.7
Aromadendrane	Aromadendrene	1437	1438	1.7	1.5	2.4	4.2	1.9	1.6	2.1	2.2	2.3	3.2	2.3	1.6
Farnesane	*Z*-β-Farnesene	1439	1440												
Humulane	α-Humulene	1450	1452	1.2	4.0	7.2	3.9	2.3	6.4	4.32	4.3	5.5	7.5	0.3	0.1
Farnesane	*E*-β-Farnesene	1453	1454												1.2
Aromadendrane	*allo*-Aromadendrene	1457	1458	0.4	0.8			0.2					0.9		
Aromadendrane	dehydro-Aromadendrane	1459	1460				2.3								
Cadinane	*Z-*Cadina-1(6),4-diene	1461	1461			1.2									
Caryophyllane	9-*epi-E*-Caryophyllene	1462	1464												
	γ-Gurjunene	1472	1475	0.2	0.5						1.6			2.1	3,2
Cadinane	γ-Muurolene	1477	1478		1.3			0.1			0.1				
Cadinane	Amorpha-4,7(11)-diene	1479	1479		1.2	0.2	0.1								
Germacrane	Germacrene D	1481	1480	0.5	7.5	4.7	5.7	5.3	2.3	7.8	1.2	1.1	4.6	1.2	4.2
Cadinane	α-Amorphene	1482	1483		1.9	0.1		tr		0.3		0.1	tr	1.3	0.1
Eremophilane	Aristolochene	1485	1487							tr	tr	tr			
Eudesmane	*Z*-Eudesma-6,11-diene (Eudesmadiene)	1488	1489	3.1	4.7	8.4	10.2	14.3	15.3	11.2	9.3	10.2	7.3	2.3	4.2
Eudesmane	β-Selinene	1493	1492	0.7	1.9			1.5		tr	0.1	tr	tr	0.1	2.3
Cadinane	γ-Amorphene	1494	1495	0.4		tr		1.0			0.9	0.1			3.4
Eremophilane	Valencene	1496	1496		2.8		0.4								
Eudesmane	α-Selinene	1498	1498					0.3		0.9	1.8	0.2	0.8	0.2	2.0
Bicyclogermacrene	Bicyclogermacrene	1499	1500	12.2	17.0	16.9	18.1	20.3	19.3	15.3	12.3	11.2	20.2	23.2	12.1
Cadinane	α-Muurolene	1502	1500		0.5	0.1				tr		0.1	tr		
Farnesane	*E,E*-α-Farnesene	1504	1505					tr							
Bisabolane	β-Bisabolene	1506	1505		0.2	0.9				tr			tr	0.1	
Cadinane	γ-Cadinene	1512	1513	0.5	1.0	1.0		0.1		tr	1.2	4.2	0.1	1.0	
Eudesmane	7-*epi-*α-Selinene	1518	1520					tr							1.2
Cadinane	δ-Cadinene	1521	1522	1.6	1.2	0.2	0.1			tr				3.3	1.3
Cadinane	Zonarene	1528	1528												tr
Cadinane	*Z-*Cadina-1,4-diene	1533	1533	1.2		0.3	tr	tr		tr		1.6	0.2	0.7	0.1
Cadinane	α-Cadinene	1537	1537	2.3		tr	0.5	tr		tr		1.2	1.2	2.2	
Eudesmane	Selina-3,7(11)-diene	1545	1545			0.1		tr						2.6	0.2
Elemane	Elemol	1548	1548	0.4						0.3					0.3
Germacrane	Germacrene B	1557	1559		2.1	1.2	2.3	5.67	1.2	1.2	3.0	2.3	7.0	5.4	2.3
Cadinane	β-Calacorene	1564	1564												
Farnesane	*E-*Nerolidol	1561	1561	17.6	22.9	6.3	5.8	4.3	4.2	3.8	4.6	4.3	5.3	10.3	15.9
Farnesane	*Z*-Nerolidol	1531	1531	0.3			tr						tr	0.1	0.2
Aromadendrane	Spathulenol	1576	1577	1.4	1.0	3.3			2.3	1.2	2.3	1.2	tr	2.1	1.4
Caryophyllane	Caryophyllene oxide	1582	1582	1.4	1.5	1.1			1.2			1.3	1.0	2.2	2.3
Aromadendrane	Viridiflorol	1592	1592	1.8	1.2	3.2	4.4		5.8	tr	2.2	1.9	0.3	3.5	3.6
Eudesmane	Rosifoliol	1602	1600	1.8	0.4								0.2		
Aromadendrane	Ledol	1601	1602	5.3	0.3	4.0	1.2		3.5	tr	4.1	1.6	0.5	1.2	2.7
Eudesmane	5-*epi*-7-*epi*-α-Eudesmol	1606	1607	0.3					tr						
Humulane	Humulene epoxide II	1608	1608	3.9	0.2				0.1		0.1			1.1	0.7
Cadinane	1,10-di-*epi*-Cubenol	1618	1618	1.0		tr			0.1		0.4			0.1	0.1
Cadinane	α-Corocalene	1620	1622	2.4										0.1	
Eudesmane	10-*epi*-γ-Eudesmol	1622	1622								2.3				
Cadinane	Muurola-4,10(14)-dien-1-β-ol	1628	1630									1.2			
Eudesmane	γ-Eudesmol	1631	1630										0.3		
Cadinane	*epi*-α-Muurolol	1640	1640	1.0	1.2	tr		tr	tr		tr	0.2	0.1		
Eudesmane	Selina-3,11-dien-6-α-ol	1642	1642											0.5	
Cadinane	α-Muurolol	1644	1644		0.7	1.7			tr	0.2	0.1	2.4	1.2	0.5	
Eudesmane	α-Eudesmol	1652	1652	0.4	0.6	2.4	3.5	tr		1.0	tr	2.9		tr	1.0
Cadinane	α-Cadinol	1652	1652	6.5	2.3	2.3	1.4	5.8	6.9	9.4	11.2	8.3	9.2	2.3	1.2
Cadinane	*Z-*Calamenen-10-ol	1660	1660										tr		
Eudesmane	7-*epi*-α-Eudesmol	1662	1662	5.6							1.0	2.9		0.10	
Caryophyllane	Caryophylla-4(12),8(13)-dien-5α-ol	1639	1639		0.2				tr	tr	tr	tr	tr	tr	
Caryophyllane	14-hydroxy-*Z*-Caryophyllene	1666	1666	1.5	0.3					tr	0.1	tr	0.7	0.1	tr
Caryophyllane	14-hydroxy-9-*epi-E*-Caryophyllene	1668	1668											2.9	tr
Cadinane	Cadalene	1675	1675	1.7							1.0				
-	undefined *m/z* 264	1677	-	0.4	0.4					0.1	1.2				
Cadinane	Amorpha-4,9-dien-2-ol	1700	1700		0.2						0.4	0.2			
Caryophyllane	Caryophyllene acetate	1701	1701									0.9			
Cadinane	Amorpha-4,9-dien-14-al	1704	1704												0.2
Octadecano	*n-*Octadecane	1801	1800								tr			0.4	0.2
Non-Oxygenated Monoterpenes				0.6	0.6	0.7	0.7	0.0	0.0	0.0	0.0	0.0	0.0	0.0	0.0
Oxygenated monoterpenes				7.9	0.1	7.5	17.0	9.7	0.0	0.0	0.0	0.0	0.0	0.3	0.1
Non-Oxygenated Sesquiterpenes				37.9	64.1	64.4	60.5	79.1	67.3	80.4	68.2	69.0	81.5	67.6	67.5
Oxygenated sesquiterpenes				50.5	33.6	24.3	16.3	10.5	24.1	15.7	30.2	29.4	18.3	27.0	29.6
Other compounds				0.0	0.1	0.0	0.0	0.0	0.0	0.0	0.1	0.0	0.0	0.4	0.2
Identified compounds in numbers				44	49	41	32	38	26	36	42	37	36	43	43
Identified compounds in relative percentage (%)				96.3	98.5	96.9	94.5	99.2	91.5	96.0	98.5	98.5	99.8	95.3	97.4
Yields (%)				0.12	0.09	0.08	0.03	0.09	0.06	0.02	0.02	0.05	0.09	0.11	0.14
GM_RO_ ^b^				−3.4	−3.2	−3.7	−4.7	−4.1	−5.6	−4.3	−3.7	−4.2	−4.4	−3.4	−3.6

RI_calc_ = Calculated Retention Index (HP-5MS column); RI_lit_ = Literature Retention index (Adams 2017); Main constituents in bold. SD = Standard Deviation. ^a^ All compounds were identified by MS and IR in accordance with experimental. ^b^ GM_RO_ = General Mixture Redox Index. tr = Trace (relative percentage value less than 0.05%).

**Table 2:** Results of circadian rhythm analysis of the essential oils obtained from leaves of *Piper gaudichaudianum* Kunth (Piperaceae) collected in March and October 2017. Yields and General Mixture Redox Index (GM_RO_) are also presented. Quantities are averaged out of three replicates.

C-Skeleton	Compounds ^a^	IR_calc_	IR_lit_		Relative Peak Area (%) ± SD															
					Rainy Season (March)								Dry Season (October)							
					6 a.m.	9 a.m.	12 p.m.	3 p.m.	6 p.m.	9 p.m.	12 a.m.	3 a.m.	6 a.m.	9 a.m.	12 p.m.	3 p.m.	6 p.m.	9 p.m.	12 a.m.	3 a.m.
Myrcane	Linalool	1093	1095								tr	tr								
Menthane	Limonene	1021	1024		tr				0.1	0.1	0.1									
Menthane	Camphor	1140	1141		0.1				0.8	0.2	0.1									
Menthane	α-Terpineol	1183	1186		0.1				1.0	1.4	0.1	0.1								
Elemane	δ-Elemene	1331	1335		0.6	3.7	3.5	3.1	0.5	0.3		6.0	0.6	3.2	3.5	3.1	0.5	0.3	9.9	6.0
Cubebane	α-Cubebene	1344	1348		tr		0.3	0.3	0.2	0.7	0.1	0.3	0.1	0.5		0.2		tr	tr	tr
Myrcane	Neryl acetate	1353	1359			1.6	2.3	3.4	0.4			0.4	2.3	3.2	1.2	0.1				1.2
Copaane	α-Copaene	1372	1374		2.2	1.6	2.1	4.1	4.4	1.3	1.6	4.3	5.1	6.3	6.8	7.1	5.4	4.3	4.2	4.3
-	Undefined *m/z* 202	1379	-		tr	2.1	1.2	0.2						0.2	0.6	0.3	tr			
Elemane	β-Elemene	1387	1389		2.1	1.2	0.3	0.3	0.9	0.6	1.2	1.1	0.3	tr	0.1	0.1	0.4	0.3	0.6	1.3
Caryophyllane	*iso-*Caryophyllene	1406	1409		0.2	0.3	0.4	1.2	1.5	0.1	tr	0.6	0.5	0.3	0.2	1.0	1.7	0.7	0.1	0.8
Caryophyllane	*E*-Caryophyllene	1416	1419		4.2	9.1	13.3	12.2	22.7	4.7	1.3	3.9	4.4	8.2	9.3	19.2	20.2	4.8	4.4	4.2
Copaane	β-Copaene	1428	1430		0.7	0.3	0.1	0.1	0.1	0.7	0.8	1.5	1.0	0.9	0.8	0.4	0.3	1.2	1.0	1.2
Aromadendrane	β-Gurjunene	1431	1434		0.5			0.3	0.1			2.0				0.2	0.1			0.3
Humulane	β-Humulene	1435	1436		1.2	2.3	3.2	3.9	4.2	0.1	tr	0.5	3.4	3.6	4.1	4.3	3.6	tr	tr	3.7
Elemane	γ-Elemene	1436	1437		1.2	1.0	0.1	0.1	tr	tr	0.4	0.4	tr					tr	tr	tr
Aromadendrane	Aromadendrene	1438	1438		2.3	1.3	1.6	2.3	3.5	1.5	0.1	tr	3.2	3.1	2.3	1.3	2.3	1.9	1.0	1.2
Humulane	α-Humulene	1450	1452		4.0	5.2	5.0	4.7	5.4	1.3	0.6	3.4	4.3	6.6	6.0	7.2	5.6	0.4	0.1	3.2
Farnesane	*E*-β-Farnesene	1452	1454										1.0							1.2
Aromadendrane	*allo*-Aromadendrene	1457	1458		1.9	0.3	0.2	0.5	0.4	1.2	1.3	1.9	1.2	1.8	2.0	2.1	2.2	0.2	0.3	2.3
Cadinane	Amorpha-4,7(11)-diene	1476	1479						0.3			0.1	0.3	0.1				0.1	0.2	tr
Germacrane	Germacrene D	1481	1480		1.5	5.6	6.0	5.3	6.7	1.1	0.5	2.1	2.3	8.3	9.3	6.3	5.3	3.1	3.5	2.3
Cadinane	α-Amorphene	1482	1483										0.3	tr		tr	0.1	2.1	1.3	1.2
Eudesmane	cis-Eudesma-6,11-diene (Eudesmadiene)	1486	1489		18.5	4.9	1.5	3.4	4.8	19.3	21.7	16.1	6.5	2.3	2.5	4.5	3.5	8.3	12.7	5.2
Eudesmane	β-Selinene	1490	1492				0.2	0.3	0.2				tr					3.2	3.6	2.3
Cadinane	γ-Amorphene	1493	1495				0.4	tr	0.1				0.4	tr		tr		0.9	1.23	4.0
Eudesmane	α-Selinene	1496	1498						0.2				0.1					0.8	1.5	1.6
Bicyclogermacrane	Bicyclogermacrene	1498	1500		15.7	19.6	19.4	19.7	19.1	13.3	14.0	14.9	13.2	28.6	26.8	18.3	18.2	11.8	10.2	12.9
Cadinane	α-Muurolene	1504	1500						tr	0.9	0.9	0.2						0.2	tr	tr
Cadinane	γ-Cadinene	1510	1513				tr	tr	0.1									0.4		
Eudesmane	7-*epi*-α-Selinene	1518	1520		0.1		0.8	0.3	0.1			tr						tr	tr	tr
Cadinane	δ-Cadinene	1523	1522		2.3	0.2	0.3	3.2	2.2	1.0	1.4	6.0	4.3	0.3	0.5	0.3	1.3	4.6	4.9	5.5
Cadinane	Zonarene	1526	1528		0.5				0.2			0.4								
Eremophilane	γ-Vetivenene	1530	1531		tr				tr			0.2								
Cadinane	*E*-Cadina-1,4-diene	1532	1533		tr				tr			0.8								
Cadinane	α-Cadinene	1535	1537										0.3							0.2
Eudesmane	Selina-3,7(11)-diene	1542	1545												0.4	0.1			0.1	
Germacrane	Germacrene B	1557	1559		0.7				0.2			0.1	0.2							0.2
Farnesane	*E*-Nerolidol	1560	1561		0.6	10.3	14.2	10.3	6.1	0.3		0.5	4.9	10.3	15.3	12.3	8.4	3.2	1.2	4.2
Cadinane	β-Calacorene	1563	1564		tr	tr	tr	tr	tr			0.8	0.2							0.1±0.0
Farnesane	*Z*-Nerolidol	1531	1531										0.3	0.1	0.7	0.2				
Aromadendrane	Spathulenol	1574	1577		7.1	3.3	4.4	4.9	4.3	8.3	10.9	5.0	9.1	2.3	1.4	1.9	3.3	10.3	15.9	9.0
Caryophyllane	Caryophyllene oxide	1579	1582		2.7	1.1	1.1	1.3	1.3	2.1	2.2									
Aromadendrane	Viridiflorol	1588	1592		4.3		1.7	2.0	1.0	0.4	1.2	1.1	1.4	1.6	1.8	1.9	1.1	1.8	2.1	1.2
Eudesmane	Rosifoliol	1598	1600		tr	tr				1.0	2.0	0.8								
Aromadendrane	Ledol	1601	1602		1.4						0.2	2.0			0.3	0.2	0.3			
Eudesmane	5-*epi*-7-*epi*-α-Eudesmol	1605	1607							0.6		0.7	0.9							tr
Humulane	Humulene epoxide II	1609	1608		tr					2.3	1.2	1.0	2.3							1.3
Cadinane	1,10-di-*epi*-Cubenol	1615	1618							1.3	1.5	1.0								
Cadinane	α-Corocalene	1623	1622			0.3	0.1	0.1	0.1	0.3	2.6									tr
Cadinane	*epi*-α-Muurolol	1638	1640			0.4	0.3	0.1		2.3	2.3									0.1
Cadinane	α-Muurolol	1642	1644							1.0	0.9	0.8	0.1							
Eudesmane	β-Eudesmol	1648	1650		1.3	3.0	4.0	4.6	0.8	0.4	0.8		0.9							
Eudesmane	α-Eudesmol	1651	1652							0.6	1.0	2.3	0.3							
Cadinane	α-Cadinol	1653	1652		12.1	2.3	1.2	0.2	2.3	14.1	15.4	14.0	9.3	2.2	1.2	4.3	6.1	19.4	10.2	9.1
Cadinane	*Z*-Calamenen-10-ol	1660	1660						tr	tr	0.1	0.3	0.3					tr	tr	0.8
Caryophyllane	14-hydroxy-*Z*-Caryophyllene	1664	1666		1.9	1.6	1.2	1.0	0.2	2.3	2.4	0.8	tr	tr	0.1	0.2	0.1	0.6	0.4	tr
Cadinane	Cadalene	1672	1675		0.2	2.0	1.3	0.4	0.3			0.3							0.1	0.3
Cadinane	Amorpha-4,9-dien-2-ol	1697	1700		0.3					2.2	2.3	0.1						tr	tr	
-	Undefined *m/z* 220	1718	-		1.2					1.1	0.3		0.4				1.2	1.3	1.9	0.9
Non-Oxygenated Monoterpenes				0,0		0.0	0.0	0.0	0.0	0.0	0.0	0.0	0.0	0.0	0.0	0.0	0.0	0.0	0.0	0.0
Oxygenated monoterpenes				0.2		1.6	2.3	3.4	2.4	1.8	0.4	0.6	2.3	3.2	1.2	0.1	0.0	0.0	0.0	1.2
Non-Oxygenated Sesquiterpenes				60.1		57.7	57.9	63.2	78.1	48.3	48.7	63.0	53.0	71.2	71.5	73.3	70.1	49.6	51.0	60.0
Oxygenated sesquiterpenes				33.1		22.1	28.2	24.5	16.2	40.5	44.8	31.0	30.4	16.6	20.9	21.0	20.8	36.8	31.9	26.6
Other compounds				0.0		0.0	0.0	0.0	0.0	0.0	0.0	0.0	0.0	0.0	0.0	0.0	0.0	0.0	0.0	0.0
Identified compounds in numbers				40		28	33	34	43	38	38	43	39	26	24	28	24	32	33	41
Identified compounds in relative percentage (%)				93.9		84.5	91.9	94.3	97.0	90.9	93.9	99.4	86.2	94.2	97.1	97.5	91.1	86.7	92.8	93.8
Yields (%)				0.23		0.19	0.10	0.14	0.13	0.17	0.12	0.10	0.14	0.11	0.09	0.11	0.12	0.15	0.16	0.13
GM_RO_ ^b^				-3.9		-4.8	-4.5	-4.5	-3.6	-3.7	-3.9	-3.7	-3.6	-5.7	-6.4	-6.0	-6.0	-4.2	-4.3	-3.6

RI_calc_ = Calculated Retention Index (HP-5MS column); RI_lit_ = Literature Retention index (Adams 2017); Main constituents in bold. SD = Standard Deviation. ^a^ All compounds were identified by MS and IR in accordance with experimental. ^b^ General Mixture Redox Index. Tr—Trace (relative percentage value less than 0.05%).

**Table 3:** Pearson’s correlation analysis between environmental abiotic variables, major compounds, chemical classes and calculated General Mixture Redox Index (GM_RO_) of the essential oils obtained from leaves of *Piper gaudichaudianum* Kunth (Piperaceae), collected from January to December 2017 (seasonality) and in the rainy (March 2017) and dry season (October 2017) (circadian rhythm).

Analyzed Variables	r								
	Relative Humidity (%)			Temperature (°C)			Radiation (KJm^−2^)		Precipitation (mm)
	Annual	March	October	Annual	March	October	March	October	Annual
Yields (%)	0.361	0.478	−0.887 **	0.084	−0.154	−0.787 **	−0.394	−0.862 **	−0.350
Bicyclogermacrene	−0.035	−0.373	0.703 *	0.228	0.347	0.588	0.855 **	0.861 **	−0.057
Eudesmadiene	−0.631 *	0.292	−0.775 *	0.366	−0.260	−0.629	−0.916 **	−0.635	−0.716 **
*E*-Caryophyllene	−0.432	−0.598	0.535	0.119	−0.311	0.724 *	0.676	0.324	−0.463
α-Cadinol	−0.751 *	0.419	−0.581	−0.209	−0.276	−0.509	−0.896 **	−0.756 *	−0.749 **
Spathulenol	0.031	0.235	−0.850 **	−0.097	−0.213	−0.826 **	−0.619	−0.766 *	0.110
*E*-Nerolidol	0.791 **	−0.097	0.911 **	−0.472	0.474	0.871 **	0.956 **	0.915 **	0.769 **
Non-Oxygenated Monoterpenes	0.735 *	-	-	−0.388	-	-	-	-	0.701 *
Oxygenated monoterpenes	0.358	−0.490	0.038	0.072	−0.029	−0.177	0.796 **	0.313	0.296
Non-Oxygenated Sesquiterpenes	−0.593 *	−0.566	0.735 *	0.625*	−0.334	0.791 *	0.328	0.762 *	−0.591 *
Oxygenated sesquiterpenes	0.480	0.533	−0.588	−0.721 **	0.008	−0.589	−0.588	−0.706 *	0.506
Other compounds	0.240	-	-	0.075	−	-	-	-	0.300
GM_RO_	−0.362	−0.0803	−0.762 **	0.520	−0.823 **	−0.809 **	−0.649	−0.776 **	−0.143

* Significant at *p* < 0.05 ** Significant at *p* < 0.01.

**Table 4:** Percentages of the carbon skeletons of the essential oils from leaves of *Piper gaudichaudianum* Kunth (Piperaceae) in the seasonality study for the period of 12 months (January to December 2017). Quantitative values are averaged from three replicates.

C-Skeleton	Percentages (%)											
	Jan	Feb	Mar	Apr	May	Jun	Jul	Aug	Sept	Oct	Nov	Dec
Aromadendrane	10.9	6.4	12.9	12.1	2.1	13.1	5.7	14.8	10.1	9.0	13.3	19.3
Bicyclogermacrane	12.2	17.0	16.9	18.1	20.3	19.3	15.3	12.3	11.2	20.2	23.2	12.1
Bisabolane	-	0.2	0.9	-	-	-	tr	-	-	tr	0.1	-
Bourbonane	-	0.1	0.1	tr	-	-	-	-	0.1	-	-	-
Cadinane	18.5	11.5	7.2	2.2	7.0	7.0	10.0	15.3	19.7	12.1	11.4	6.5
Caryophyllane	6.1	10.8	10.1	6.9	7.7	11.8	12.5	5.6	8.7	10.4	7.0	5.5
Camphane	0.1	0.1	5.0	5.2	5.1	-	-	-	-	-	-	tr
Copaane	-	1.9	1.6	-	5.7	-	9.6	8.5	7.2	8.2	2.7	5.3
Cubebane	-	0.2	tr	-	1.2	-		0.4	5.6	3.4	1.2	2.9
Elemane	3.6	1.3	9.0	5.6	10.2	10.5	10.6	12.4	5.4	3.2	6.3	9.9
Eremophilane	Tr	2.8	tr	0.4	-	-	tr	tr	tr	-	-	-
Eudesmane	11.9	7.6	10.8	13.7	16.1	15.4	12.1	14.5	16.2	8.6	5.8	11.0
Farnesane	17.9	22.9	6.3	5.8	4.3	4.2	3.8	4.6	4.3	5.3	10.3	17.3
Germacrane	0.5	9.9	5.9	8.01	11.0	3.6	9.0	4.2	3.4	11.6	6.7	6.5
Guaiane	0.2	0.5	-	-	-	-	-	1.6	-	-	2.1	3.2
Humulane	5.1	4.2	7.2	3.9	2.3	6.5	6.6	4.4	5.5	7.6	1.4	0.8
Menthane	0.6	-	1.2	6.3	2.1	tr	-	-	-	-	-	tr
Myrcane	6.3	-	1.2	5.8	2.8	tr	0.5	-	-	-	0.3	-
Pinane	0.6	0.7	0.7	0.4	-	-	-	-	-	-	-	-

Tr—Trace (percentage value less than 0.05%).

**Table 5:** Percentages of the carbon skeletons of the essential oil components from leaves of *Piper gaudichaudianum* Kunth (Piperaceae) in the circadian study, during the Rainy (R, March) and Dry (D, October) season, from 12 a.m. to 12 p.m.

C-Skeleton	Percentages (%)							
	Rainy Season (March)							
	6 a.m.	9 a.m.	12 p.m.	3 p.m.	6 p.m.	9 p.m.	12 a.m.	3 a.m.
Aromadendrane	17.4	5	7.9	10.1	9.3	11.5	13.8	12.1
Bicyclogermacrane	15.8	19.6	19.4	19.7	19.1	13.3	14	14.9
Cadinane	15.5	5.3	3.8	4.2	5.7	23.3	27.5	25
Camphane	0.1	-	-	-	0.8	0.2	0.1	-
Caryophyllane	9.1	12.2	16	15.8	25.7	9.3	6	5.3
Copaane	2.9	2	2.2	4.3	4.5	1.9	2.3	5.8
Cubebane	0		0.3	0.3	0.2	0.7	0.1	0.3
Elemane	3.9	5.4	3.9	3.5	1.4	1	1.7	7.6
Eremophilane	-	-	-	-	-	-	-	0.2
Eudesmane	19.9	8	6.5	8.7	6.1	21.9	25.4	19.8
Farnesane	0.6	10.3	14.2	10.3	6.1	0.3	0	0.5
Germacrane	2.1	5.7	6	5.3	6.9	1.1	0.5	2.2
Humulane	5.3	7.5	8.2	8.5	9.6	3.8	1.9	5
Menthane	0.2	-	-	-	1.9	1.8	0.3	0.1
Myrcane	0	1.6	2.3	3.5	0.4	0	0	0.5
**Dry season (October)**								
Aromadendrane	15	8.8	7.8	7.7	9.4	14.2	19.3	14
Bicyclogermacrane	13.2	28.6	26.8	18.3	18.2	11.8	10.2	12.9
Cadinane	15.7	2.7	1.7	4.7	7.5	27.8	18.1	21.4
Camphane	-	-	-	-	-	-	-	-
Caryophyllane	4.9	8.6	9.6	20.4	22	6	5	5.1
Copaane	6.1	7.2	7.5	7.6	5.8	5.6	5.2	5.6
Cubebane	0.1	0.5		0.2		0	0	0
Elemane	0.9	3.2	3.6	3.2	0.9	0.7	10.4	7.4
Eremophilane	-	-	-	-	-	-	-	-
Eudesmane	8.7	2.3	2.9	4.6	3.5	12.3	17.8	9.2
Farnesane	6.2	10.4	16.1	12.5	8.4	3.2	1.2	5.5
Germacrane	2.6	8.3	9.3	6.3	5.3	3.1	3.5	2.5
Humulane	10.1	10.2	10.1	11.6	8.9	0.5	0.1	8.2
Menthane	-	-	-	-	-	-	-	-
Myrcane	2.3	3.2	1.2	0.1	-	-	-	1.2

## Data Availability

Not applicable.

## Supplementary Materials

The supplementary data available online at https://www.mdpi.com/article/10.3390/plants10102116/s1 Table S1. Simple correlation between the main constituents of the essential oils from leaves of Piper gaudichaudianum Kunth collected for seasonality study. Table S2. Simple correlation between the main constituents of the essential oils from leaves of Piper gaudichaudianum Kunth collected for the circadian rhythm study. Table S3. Database of literature on compounds and carbon skeletons of Piper gaudichaudianum Kunth leaves’ essential oils used to determine chemophenetic variations. Scheme S1. Biosynthetic map of terpene carbon skeleton types based on essential oils from leaves of from Piper gaudichaudianum Kunth of this study and database from literature. Scheme S2. Biosynthetic map of terpene carbon skeleton types based on the essential oils compounds from leaves of Piper gaudichaudianum Kunth (Piperaceae) for seasonality studies. Scheme S3. Biosynthetic map of terpene carbon skeleton types based on the essential oils compounds from leaves of Piper gaudichaudianum Kunth (Piperaceae) for circadian rhythm studies. Figure S1. Structures of terpenic derivatives (monoterpenoids and sesquiterpenes) and non-terpenic hydrocarbons identified in the essential oils of Piper gaudichaudianum Kunth with their respective oxidation numbers (NOX) and the values of the oxidation steps (OS) of terpene-type chemical precursors. Figure S2. Climatic data of Rio de Janeiro City (Brazil) during the collections of Piper gaudichaudianum Kunth leaves. Data collected from reference INMET (2020). Monthly averages of temperature, precipitation, and relative humidity from January to December 2017 (A). Ombrothermal diagram from January to December 2017 (B). Data of temperature, relative humidity, and radiation from the leaves´ collection time for the circadian study in March (C) and October 2017 (D).
