# Supplementary material for: Advanced Chemophenetic Analysis of Essential Oil from Leaves of Piper gaudichaudianum Kunth (Piperaceae) Using a New Reduction-Oxidation Index to Explore Seasonal and Circadian Rhythms
Source: Plants (Basel). 2021 Oct 6;10(10):2116. doi: 10.3390/plants10102116 (PMC8540104; doi:10.3390/plants10102116)
Supplement: Supplementary file 1 [file plants-10-02116-s001.zip › plants-1403457-supplementary.pdf]

Supplementary Material

Table S1. Simple correlation between the main constituents of the essential oils from leaves of *Piper gaudichaudianum* Kunth collected for seasonality study.

Compound	α-Cadinol	Bicyclogermacrene	*E*-Nerolidol	*E*-Caryophyllene	Eudesmadiene	Non-oxygenated Monoterpene	Oxygenated monoterpenes	Non-oxygenated Sesquiterpenes	Oxygenated sesquiterpenes
Bicyclogermacrene	-0.269								
(*E*)-Nerolidol	-0.508	-0.192							
E-Caryophyllene	0.294	0.340	-0.454						
Eudesmadiene	0.396	0.125	-0.746*	0.635*					
Non-oxygenated Monoterpene	-0.536	-0.057	0.457	-0.036	-0.278				
Oxygenated monoterpenes	-0.402	0.152	-0.111	-0.115	0.179	0.6137*			
Non-oxygenated Sesquiterpenes	0.318	0.353	-0.540	0.571	0.453	-0.653*	-0.380		
Oxygenated sesquiterpenes	-0.066	-0.532	0.679*	-0.598*	-0.644*	0.350	-0.165	-0.828**	
Other compounds	-0.515	0.330	0.391	-0.492	-0.632*	-0.160	-0.268	-0.026	0.145

*Significant at $p < 0.05$ **Significant at $p < 0.01$

Tabela S2. Simple correlation between the main constituents of the essential oils from leaves of *Piper gaudichaudianum* Kunth collected for the circadian rhythm study.

Rainy Season (March)								
Compound	Oxygenated monoterpenes	Non-oxygenated Sesquiterpenes	Oxygenated sesquiterpenes	Bicyclogermacrene	Eudesmadiene	*E*-Caryophyllene	α-Cadinol	Spathulenol
Non-oxygenated Sesquiterpene	0.366							
Oxygenated sesquiterpenes	-0.582	-0.854*						
Bicyclogermacrene	0.698	0.594	-0.870*					
Eudesmadiene	-0.798*	-0.554	0.843*	-0.963**				
E-Caryophyllene	0.723*	0.800*	-0.850*	0.774*	-0.810*			
α-Cadinol	-0.808*	-0.536	0.836*	-0.981**	0.980**	-0.806*		
Spathulenol	-0.531	-0.654	0.906**	-0.817*	0.843*	-0.656	0.787*	
E-Nerolidol	0.736*	0.277	-0.646	0.907*	-0.946**	0.628	-0.932**	-0.716*
**Dry Season (October)**								
Compound	Oxygenated monoterpenes	Non-oxygenated Sesquiterpenes	Oxygenated sesquiterpenes	Bicyclogermacrene	Eudesmadiene	*E*-Caryophyllene	α-Cadinol	Spathulenol
Non-oxygenated Sesquiterpene	0.168							
Oxygenated sesquiterpenes	-0.381	-0.954**						
Bicyclogermacrene (%)	0.524	0.818*	-0.850**					
Eudesmadiene (%)	-0.446	-0.843*	0.822*	-0.812*				
E-Caryophyllene (%)	-0.360	0.744*	-0.610	0.337	-0.495			
α-Cadinol	-0.413	-0.857*	0.923**	-0.795*	0.680	-0.463		
Spathulenol	-0.271	-0.926**	0.851*	-0.842*	0.944**	-0.673	0.723*	
E-Nerolidol	0.211	0.898**	-0.806*	0.861**	-0.823**	0.584	-0.797*	-0.932**

* Significant $p < 0.05$ ** Significant $p < 0.01$

Table S3. Database of literature on compounds and carbon skeletons of *Piper gaudichaudianum* Kunth leaves' essential oils used to determine chemophenetic variations.

C-Skeleton	Compound	RO	RS1-1	RS1-2	RS1-3	RS1-4	RS1-5	RS1-6	RS1-7	RS1-8	RS1-9	RS1-10	RS1-11	RS1-12	RS1-13	RS1-14	RS1-15
Alkylbenzene (C_3-C_6)	Dillapiole		63.6	63.4	66.7	57.8	68.4	62.7	70.5	60.5	66.6	61.6	65.2	59.2	69.2	68.8	68.1
Aromadendrane	Aromadendrene	15.6	0.4	2.5	2.4	3.5	2.6		2.4	1.6		2	1.5		0.2	1.7	2
Aromadendrane	Spathulenol		0.2	0.2		0.2	0.9	2.1	0.2	1.8	2.1	1.2	1.9		1.6		0.2
Aromadendrane	Viridiflorol	27.5															
Bourbonane	β-Bourbonene		0.5	0.4		0.5			0.5						0.3	0.4	0.3
Cadinane	γ-Muurolene	4.35															
Cadinane	δ-Cadinol														0.8	0.5	0.4
Cadinane	γ-Cadinol					0.2									0.3	0.6	0.2
Cadinane	*t*-Muurolol									0.8							
Caryophyllane	*E*-Caryophyllene		3.7	4.2	4.0	5.0	3.5	3.8	3.8	4.6	3.9	4.2	3.8	4.9	4	3.1	3.7
Caryophyllane	Caryophyllene oxide					0.3									0.6	0.7	0.5
Chromane	Eupatoriochromene	3.61															
Copaane	α-Copaene		0.4	0.4		0.5									0.3	0.3	0.3
Copaane	β-copaene		0.6												0.4	0.4	0.4
Cubebane	δ-Cadinene		0.5	1.3	1.6	3.7		2.7		2.3		2.8		1.9	1.7	1.3	4
Eremophilane	Aristolene	3.88															
Eudesmane	α-Selinene	3.9										1.3		1.5			
Eudesmane	β-Selinene	10.5															
Eudesmane	Selin-11-en-4-α-ol	8.48															
Farnesane	*E*-Nerolidol		0.9	0.9		1.1		3.0					3	2.9			
Germacrane	Germacrene D		2.1														
Germacrane	Germacrene B		6.9	4.7	5.8	5.9	6.1	6.9	5	7.4	5.1	6.1	5.9	5.4	4.4	4.4	1.2
Guiane	α-Bulnesene					1.6									1.9	1.1	1.6
Humulane	α-Humulene		11.8	13.3	12	14.3	10.2	8.2	7.9	10.1	9.7	9.3	9.3	11.2	9.6	8.4	10.2
Humulane	Epoxy Humulene II		0.6	0.4													
Humulane	Humulene oxide	4.15															
Ishwarane	Ishwarene	10.0	3.9	4.3	3.3	1.4	2.5	4.1	2.8	3.7	5	4.2	3.8	6.4	1.3	3.7	1.9
**Chemical classes**	**Monoterpene hydrocarbons**	2.4	0.0	0.0	0.0	0.0	0.0	0.0	0.0	0.0	0.0	0.0	0.0	0.0	0.0	0.0	0.0

	**Oxygenated monoterpenes**	0.0	0.0	0.0	0.0	0.0	0.0	0.0	0.0	0.0	0.0	0.0	0.0	0.0	0.0	0.0	0.0
	**Non-oxygenated Sesquiterpenes**	48.2	30.8	31.1	29.1	36.4	24.9	25.7	22.4	29.7	23.7	29.9	24.3	31.3	24.1	24.8	25.6
	**Oxygenated sesquiterpenes**	40.1	1.7	1.5	0.0	1.8	0.9	5.1	0.2	2.6	2.1	1.2	4.9	2.9	3.3	1.8	1.3
	**Arylpropanoids (C_6-C_3)**	0.0	63.6	63.4	66.7	57.8	68.4	62.7	70.5	60.5	66.6	61.6	65.2	59.2	69.2	68.8	68.1
	**Benzyl benzoate (C_6-C_6)**	0.0	0.0	0.0	0.0	0.0	0.0	0.0	0.0	0.0	0.0	0.0	0.0	0.0	0.0	0.0	0.0
	**Eupatoriochromene**	3.6	0.0	0.0	0.0	0.0	0.0	0.0	0.0	0.0	0.0	0.0	0.0	0.0	0.0	0.0	0.0
	**Other compounds**	0.0	0.0	0.0	0.0	0.0	0.0	0.0	0.0	0.0	0.0	0.0	0.0	0.0	0.0	0.0	0.0
**C-Skeleton**	**Acorane**	0.0	0.0	0.0	0.0	0.0	0.0	0.0	0.0	0.0	0.0	0.0	0.0	0.0	0.0	0.0	0.0
	**Alkylbenzene (C_6-C_3)**	0.0	63.6	63.4	66.7	57.8	68.4	62.7	70.5	60.5	66.6	61.6	65.2	59.2	69.2	68.8	68.1
	**Aromadendrane**	43.05	0.6	2.7	2.4	3.7	3.5	2.1	2.6	3.4	2.1	3.2	3.4	0	1.8	1.7	2.2
	**Bergamotane**	0.0	0.0	0.0	0.0	0.0	0.0	0.0	0.0	0.0	0.0	0.0	0.0	0.0	0.0	0.0	0.0
	**Bicyclogermacrene**	0.0	0.0	0.0	0.0	0.0	0.0	0.0	0.0	0.0	0.0	0.0	0.0	0.0	0.0	0.0	0.0
	**Bisabolane**	0.0	0.0	0.0	0.0	0.0	0.0	0.0	0.0	0.0	0.0	0.0	0.0	0.0	0.0	0.0	0.0
	**Bourbonane**	0	0.5	0.4	0.0	0.5	0.0	0.0	0.5	0.0	0.0	0.0	0.0	0.0	0.3	0.4	0.3
	**Benzyl benzoate (C_6-C_6)**	0.0	0.0	0.0	0.0	0.0	0.0	0.0	0.0	0.0	0.0	0.0	0.0	0.0	0.0	0.0	0.0
	**Cadinane**	4.35	0.0	0.0	0.0	0.2	0.0	0.0	0.0	0.8	0.0	0.0	0.0	0.0	1.1	1.1	0.6
	**Carane**	0.0	0.0	0.0	0.0	0.0	0.0	0.0	0.0	0.0	0.0	0.0	0.0	0.0	0.0	0.0	0.0
	**Caryophyllane**	0.0	3.7	4.2	4.0	5.3	3.5	3.8	3.8	4.6	3.9	4.2	3.8	4.9	4.6	3.8	4.2
	**Chromane**	3.61	0.0	0.0	0.0	0.0	0.0	0.0	0.0	0.0	0.0	0.0	0.0	0.0	0.0	0.0	0.0
	**Copaane**	0.0	1	0.4	0.0	0.5	0.0	0.0	0.0	0.0	0.0	0.0	0.0	0.0	0.7	0.7	0.7
	**Cubebane**	0	0.5	1.3	1.6	3.7	0.0	2.7	0.0	2.3	0.0	2.8	0.0	1.9	1.7	1.3	4.0
	**Daucane**	0.0	0.0	0.0	0.0	0.0	0.0	0.0	0.0	0.0	0.0	0.0	0.0	0.0	0.0	0.0	0.0
	**Elemane**	0.0	0.0	0.0	0.0	0.0	0.0	0.0	0.0	0.0	0.0	0.0	0.0	0.0	0.0	0.0	0.0
	**Eremophilane**	3.88	0.0	0.0	0.0	0.0	0.0	0.0	0.0	0.0	0.0	0.0	0.0	0.0	0.0	0.0	0.0
	**Eudesmane**	22.89	0.0	0.0	0.0	0.0	0.0	0.0	0.0	0.0	0.0	1.3	0.0	1.5	0.0	0.0	0.0
	**Farnesane**	0.0	0.9	0.9	0.0	1.1	0.0	3.0	0.0	0.0	0.0	0.0	3.0	2.9	0.0	0.0	0.0
	**Germacrane**	0.0	9.0	4.7	5.8	5.9	6.1	6.9	5	7.4	5.1	6.1	5.9	5.4	4.4	4.4	1.2
	**Guiane**	0.0	0.0	0.0	0.0	1.6	0.0	0.0	0.0	0.0	0.0	0.0	0.0	0.0	1.9	1.1	1.6
	**Hidrocarbonetes**	0.0	0.0	0.0	0.0	0.0	0.0	0.0	0.0	0.0	0.0	0.0	0.0	0.0	0.0	0.0	0.0

**Himalachane**	0.0	0.0	0.0	0.0	0.0	0.0	0.0	0.0	0.0	0.0	0.0	0.0	0.0	0.0	0.0	0.0
**Humulane**	4.15	12.4	13.7	12	14.3	10.2	8.2	7.9	10.1	9.7	9.3	9.3	11.2	9.6	8.4	10.2
**Ishwarane**	10.0	3.9	4.3	3.3	1.4	2.5	4.1	2.8	3.7	5.0	4.2	3.8	6.4	1.3	3.7	1.9
**Longifolane**	0.0	0.0	0.0	0.0	0.0	0.0	0.0	0.0	0.0	0.0	0.0	0.0	0.0	0.0	0.0	0.0
**Longipinane**	0.0	0.0	0.0	0.0	0.0	0.0	0.0	0.0	0.0	0.0	0.0	0.0	0.0	0.0	0.0	0.0
**Menthane**	0.0	0.0	0.0	0.0	0.0	0.0	0.0	0.0	0.0	0.0	0.0	0.0	0.0	0.0	0.0	0.0
**Myrcane**	1.29	0.0	0.0	0.0	0.0	0.0	0.0	0.0	0.0	0.0	0.0	0.0	0.0	0.0	0.0	0.0
**Pinane**	1.14	0.0	0.0	0.0	0.0	0.0	0.0	0.0	0.0	0.0	0.0	0.0	0.0	0.0	0.0	0.0
**Thujopsane**	0.0	0.0	0.0	0.0	0.0	0.0	0.0	0.0	0.0	0.0	0.0	0.0	0.0	0.0	0.0	0.0

Reference: RO (Morais et al. 2007); RS1 - 1 to 16 (Schindler et al. 2017); RS2 (Von Poser et al. 1994); RS3 (Péres et al. 2009); RS4 (Sperotto et al. 2013); SC (Chaaban et al. 2018); SP1- 1 and 2 (Andrade et al. 1998); PR1 (Krinski et al. 2016); PR2 (Bernuci et al. 2016); PR3 - 1 and 2 (Krinski et al. 2018); PR4 (Silva et al. 2019); PR5 - 1 and 2 (Quiqui et al. 2019); PR6 (Souza et al. 2020) and; PR7 (Souza et al. 2020).

Table S3. *Continuation*

C-Skeleton	Compound	RS1 - 16	RS2	RS3	RS4	SC	SP1 - 1	SP1 - 2	PR1	PR2	PR3 - 1	PR3 - 2	PR4	PR5 - 1	PR5 - 2	PR6	PR7
Acorane	α-Acorenol							0.6									
Alkylbenzene (C3-C6)	Dillapiole	64.7															
Alkylbenzene (C3-C6)	Eugenol			0.1													
Alkylbenzene (C3-C6)	Myristicin															15.19	
Aromadendrane	Aromadendrene	2.1	2.2	2.7	2.92		2.3	-						1.44	0.37		
Aromadendrane	α-Gurjunene		< 0.01	0.2	0.14									0.54	0.44		
Aromadendrane	Alloaromadendrene		7.7	3.1	3.28		0.6	4.3		2.3							
Aromadendrane	γ-Gurjunene			4.3	0.23					2.9							
Aromadendrane	β-Gurjunene			0.4										4.94	5.13		
Aromadendrane	Spathulenol	1.4	0.7	1.8	1.97		4.5	3.8		1.4				3.2	0.82		
Aromadendrane	*allo*-Aromadendrene oxide				0.00	1.1							2.47				2.47
Aromadendrane	Ledol				0.04												
Aromadendrane	Globulol		0.5		0.16		2.9	3.1	10.3					0.1			
Aromadendrane	Viridiflorol			0.6	0.18									0.21	0.2		
Bergamotane	*E*-α-Bergamotene				0.23												
Bicyclogermacrane	Bicyclogermacrene		1.2	7.4	13.16					4.4	5.12	6.39	2.57	4.15	4.81	4.63	2.57
Bisabolane	α-Zingiberene					2.5											
Bisabolane	α-Santalene				0.33												
Bisabolane	β-Santalene				1.08												
Bisabolane	γ-Curcumene				0.12												
Bisabolane	β-Curcumene				0.52												
Bisabolane	γ-*E*-bisabolene				0.21												
Bisabolane	α-*Z*-Bisaboleno																
Bisabolane	*Z*-Calamenen-10-ol					1.5											
Bisabolane	*E*-Calamenen-10-ol					3.1											
Bisabolane	α-Bisabolol				0.02												
Bisabolane	*Z*-α-*trans*-Bergamotol				0.09												
Bisabolane	*epi*-α-bisabolol													1.9	1.38		

Bourbonane	β-Bourbonene	0.4		0.2												
C_6C_6	Benzyl benzoate							0.2	2.5							
Cadinane	α-Amorphene					1.2										
Cadinane	α-Calacorene			0.1	0.16	3.5						3.5	0.2	0.58		3.5
Cadinane	β-Calacorene					1.9						1.98				1.98
Cadinane	*E*-Muurola-3.5-diene				0.06											
Cadinane	γ-Muurolene				0.85			2.3					0.6	0.56		
Cadinane	α-Muurolene			0.3	0.73								4.0	4.98		
Cadinane	δ-Amorphene				0.21											
Cadinane	γ-Cadinene		0.4	0.8	1.27		1.1	1.6					3.8	4.05		
Cadinane	Zonarene				0.01											
Cadinane	α-Cadinene				0.15								0.3	0.37		
Cadinane	Cadalene									33.73						
Cadinane	*Z*-Cadina-1(6),4-diene												0.3	0.31		
Cadinane	*E*-Cadina-1,4-diene												0.6	0.83		
Cadinane	1.10-di-*epi*-cubenol				0.09			0.3					0.7	0.77		
Cadinane	α-Muurolol			0.3	0.02								1.9	2.17		
Cadinane	α-Cadinol		0.2		0.26		4.3	1.2	3.3				1.3	1.31		
Cadinane	1 -*epi*-Cubenol						0.7			24.22			1.2	1.59	25.05	
Cadinane	*epi*-α-Cadinol							1.1	1.6							
Cadinane	*epi*-α-Muurolol						1.2						1.9	2.21		
Cadinane	Cubebol								1.5							
Carane	δ-2-Carene			0.1												
Carane	*p*-Cymene			0.1					1.2							
Carane	δ-3-Carene			0.1					5.9							
Caryophyllane	*E*-Caryophyllene	4.3	17.4	8.9	7.51	3.7	12.1	19.3	1.7	4.31	17.84	3.75	7.4	7.25	3.71	3.75
Caryophyllane	*Z*-Cariophyllene					5.2										
Caryophyllane	Caryophyllene oxide		0.7	1.6	0.01							1.96	3.1	3.69		1.96
Caryophyllane	Caryophylla-4(14),8(15)-dien-5-α-ol				0.15											
Caryophyllane	Caryophile-4(12),8(13)-dien-5β-ol												0.2	1.2		
Copaane	α-Copaene	0.4	0.5	0.5	0.01	4.3		0.5				4.36	3.2			4.36
Copaane	β-copaene				0.2											

Copaane	α-Ylangene			tr													
Copaane	β-Capaen-4-α-ol					2											
Copaane	β–Copaen–4–α-ol										2.06		2.06				2.06
Copaane	α-Copaen-11-ol													0.2	0.14		
Cubebane	δ-Cadinene	1.5	0.8	1.2	1.47	9.3	2.4	3.5		45.3	6.86		9.39	5.6	7.12		9.39
Cubebane	Amorpha-4.7(11)-diene					2.8							2.86				2.86
Cubebane	α-Cubebene			0.2				0.2						0.6			
Daucane	Dauca-5.8-diene					2.4								5.2	6.26		
Elemane	γ-Elemene				0.06	6.1				5.4			5.24				5.24
Elemane	β-Elemene		tr	1.7	0.71		2.1	0.5		3.5			6.1				6.1
Elemane	δ-Elemene			0.2													
Elemane	Elemol			0.1	0.27												
Elemane	Shyobunol									1.2							
Eremophilane	4.5-di-*epi*-Aristolochene					1.2							1.27				1.27
Eremophilane	Aristolene				0.02												
Eremophilane	4–*epi*–*cis*-dihydro-Agarofuran												2.99				2.99
Eudesmane	α-Selinene		2.1		2.67	2.9	16.6	8.9									
Eudesmane	δ-Selinene			0.6		1.6							1.65	0.3	0.36		1.65
Eudesmane	Drima-7.9(11)-diene				0.34												
Eudesmane	β-Selinene		2	4.8	3.43		15.7	3.7						0.1	0.36		
Eudesmane	7-Epi-α-Selinene				0.16		0.4										
Eudesmane	Selina-3.7(1l)-diene						0.5										
Eudesmane	Intermedeol				0.45												
Eudesmane	Selin-11-en-4-α-ol									1.2							
Eudesmane	5-*epi*-7-*epi*-α-Eudesmol								13.3								
Eudesmane	β-Eudesmol			0.2										0.7	0.76		
Eudesmane	Eudesm-7(11)-en-4-ol															28.4	
Eudesmane	Rosifoliol													0.5	0.51		
Eudesmane	γ-Eudesmol													0.6	0.61		
Farnesane	*E.E*-α-Farnesene			tr	1.31	1.9							1.92	0.3	0.48		1.92
Farnesane	*E*-Nerolidol		4.1	22.4	22.06	3.2	2.7	2.8			4.02	5.03	3.26	6.1	7.11	5.31	3.26
Farnesane	*Z.E*-Farnesol				0.12												
Farnesane	2-Sesquilavandulol						0.4	1.6									

Farnesane	2-*Z*-6-*E*-Farnesol																
Germacrane	Germacrene D		1.3	2.2	0.03		4.7			1.7			1.2	1.4	1.73		1.2
Germacrane	Germacrene B	4.2			0.37	21.5	1.1						21.53	0.4	0.21		21.53
Germacrane	Hedycaryol					1.1											
Germacrane	Germacrene A			1.5	0.12			0.9		2.6							
Guiane	α-Bulnesene																
Guiane	α-Guaiene			0.3					8.3								
Guiane	6.9-Guaiadiene													0.2	0.18		
Hidrocarbonetes	3*Z*-hexenol													0.3			
Hidrocarbonetes	6-Methyl-5-hepten-2-one													0.8			
Himalachane	α-Hirnachalene							3.2									
Humulane	α-Humulene	10.9	37.5	16.5	21.3	3.6	13.3	29.2		2.2			3.7	2.3	1.4		3.67
Humulane	Epoxy Humulene II			2.5	2.3			2.8						0.9	0.51		
Humulane	Humulene oxide		1.1														
Ishwarane	Ishwarene	1.9															
Longifolane	Longifolol									1.2							
Longipinane	Longipinanol								19.1								
Menthane	Limonene		0.5	0.3	0.1		0.5	0.5						0.7			
Menthane	α-Phellandrene			0.1													
Menthane	α-Terpinene			0.1													
Menthane	γ-Terpinene			0.1													
Menthane	Terpinolene			0.1													
Menthane	α-Terpineol			0.1	0.01												
Myrcane	Myrcene		0.3	0.2										0.3			
Myrcane	*Z*-β-Ocimene		0.4											0.2			
Myrcane	*E*-β-Ocimene		0.6	0.2													
Myrcane	Linalool		4.8	0.3	0.04												
Myrcane	Geranyl acetate													5.3	5.7		
Pinane	α-Pinene		2.0	1.9		2.1						9.7	2.2	5.1			2.2
Pinane	β-Pinene		5.6	1.2								13.2		6.6			
Thujopsane	*Z*-dihydro-Mayurone					1.9											
**Chemical classes**	**Monoterpene hydrocarbons**	0.0	9.4	4.5	0.1	2.1	0.5	0.5	0.0	7.1	0.0	22.8	2.2	12.9	0.0	0.0	2.2
	**Oxygenated monoterpenes**	0.0	4.8	0.4	0.1	0.0	0.0	0.0	0.0	0.0	0.0	0.0	0.0	5.3	5.7	0.0	0.0

	**Sesquiterpenes hydrocarbons**	25.7	73.1	58.1	65.4	76.7	72.9	78.1	8.3	72.0	50.0	24.2	69.7	47.9	47.7	8.3	69.7
	**Oxygenated sesquiterpenes**	1.4	7.3	29.5	28.2	12.8	16.7	17.3	42.7	11.4	30.3	5.0	12.7	24.6	25.0	58.8	12.7
	**Arylpropanoids (C_6-C_3)**	64.7	0.0	0.1	0.0	0.0	0.0	0.0	0.0	0.0	0.0	0.0	0.0	0.0	0.0	15.2	0.0
	**Benzyl benzoate (C_6-C_6)**	0.0	0.0	0.0	0.0	0.0	0.0	0.2	0.0	2.5	0.0	0.0	0.0	0.0	0.0	0.0	0.0
	**Eupatoriochromene**	0.0	0.0	0.0	0.0	0.0	0.0	0.0	0.0	0.0	0.0	0.0	0.0	0.0	0.0	0.0	0.0
	**Other compounds**	0.0	5.1	0.0	0.0	7.1	0.0	0.0	0.0	0.0	0.0	0.0	0.0	1.1	0.0	0.0	0.0
**C-Skeleton**	**Acorane**	0.0	0.0	0.0	0.0	0.0	0.0	0.6	0.0	0.0	0.0	0.0	0.0	0.0	0.0	0.0	0.0
	**Alkylbenzene (C_3-C_6)**	64.7	0.0	0.1	0.0	0.0	0.0	0.0	0.0	0.0	0.0	0.0	0.0	0.0	0.0	15.2	0.0
	**Aromadendrane**	3.5	11.1	13.1	8.9	1.1	10.3	11.2	10.3	6.6	0.0	0.0	2.5	10.4	7.0	0.0	2.5
	**Bergamotane**	0.0	0.0	0.0	0.2	0.0	0.0	0.0	0.0	0.0	0.0	0.0	0.0	0.0	0.0	0.0	0.0
	**Bicyclogermacrene**	0.0	1.2	7.4	13.2	0.0	0.0	0.0	0.0	4.4	5.1	6.4	2.6	4.2	4.8	4.6	2.6
	**Bisabolane**	0.0	0.0	0.0	2.4	7.1	0.0	0.0	0.0	0.0	0.0	0.0	0.0	1.9	1.4	0.0	0.0
	**Bourbonane**	0.4	0.0	0.2	0.0	0.0	0.0	0.0	0.0	0.0	0.0	0.0	0.0	0.0	0.0	0.0	0.0
	**Benzyl benzoate (C_6-C_6)**	0.0	0.0	0.0	0.0	0.0	0.0	0.2	0.0	2.5	0.0	0.0	0.0	0.0	0.0	0.0	0.0
	**Cadinane**	0.0	0.6	1.5	3.8	6.6	7.3	6.5	0.0	6.4	58.0	0.0	5.5	16.8	19.7	25.1	5.5
	**Carane**	0.0	0.0	0.3	0.0	0.0	0.0	0.0	0.0	7.1	0.0	0.0	0.0	0.0	0.0	0.0	0.0
	**Caryophyllane**	4.3	18.1	10.5	7.7	8.9	12.1	19.3	0.0	1.7	4.3	17.8	5.7	10.7	12.1	3.7	5.7
	**Chromane**	0.0	0.0	0.0	0.0	0.0	0.0	0.0	0.0	0.0	0.0	0.0	0.0	0.0	0.0	0.0	0.0
	**Copaane**	0.4	0.5	0.5	0.2	6.3	0.0	0.5	0.0	0.0	2.1	0.0	6.4	3.3	0.1	0.0	6.4
	**Cubebane**	1.5	0.8	1.4	1.5	12.1	2.4	3.7	0.0	45.3	6.9	0.0	12.3	6.2	7.1	0.0	12.3
	**Daucane**	0.0	0.0	0.0	0.0	2.4	0.0	0.0	0.0	0.0	0.0	0.0	0.0	5.2	6.3	0.0	0.0
	**Elemane**	0.0	0.0	2.0	1.0	6.1	2.1	0.5	0.0	10.1	0.0	0.0	11.3	0.0	0.0	0.0	11.3
	**Eremophilane**	0.0	0.0	0.0	0.0	1.2	0.0	0.0	0.0	0.0	0.0	0.0	3.0	0.0	0.0	0.0	3.0
	**Eudesmane**	0.0	4.1	5.6	7.1	4.5	33.2	12.6	13.3	1.2	0.0	0.0	1.7	2.2	2.6	28.4	1.7
	**Farnesane**	0.0	4.1	22.4	23.5	5.1	3.1	4.4	0.0	0.0	4.0	5.0	5.2	6.5	7.6	5.3	5.2
	**Germacrane**	4.2	1.3	3.7	0.5	22.6	5.8	0.9	0.0	4.3	0.0	0.0	22.7	1.8	1.9	0.0	22.7
	**Guiane**	0.0	0.0	0.3	0.0	0.0	0.0	0.0	8.3	0.0	0.0	0.0	0.0	0.2	0.2	0.0	0.0
	**Hidrocarbonetes**	0.0	0.0	0.0	0.0	0.0	0.0	0.0	0.0	0.0	0.0	0.0	0.0	1.1	0.0	0.0	0.0
	**Himalachane**	0.0	0.0	0.0	0.0	0.0	0.0	3.2	0.0	0.0	0.0	0.0	0.0	0.0	0.0	0.0	0.0
	**Humulane**	10.9	38.6	19.0	23.6	3.6	13.3	32.0	0.0	2.2	0.0	0.0	3.7	3.2	1.9	0.0	3.7
	**Ishwarane**	1.9	0.0	0.0	0.0	0.0	0.0	0.0	0.0	0.0	0.0	0.0	0.0	0.0	0.0	0.0	0.0
	**Longifolane**	0.0	0.0	0.0	0.0	0.0	0.0	0.0	0.0	1.2	0.0	0.0	0.0	0.0	0.0	0.0	0.0
	**Longipinane**	0.0	0.0	0.0	0.0	0.0	0.0	0.0	19.1	0.0	0.0	0.0	0.0	0.0	0.0	0.0	0.0

**Menthane**	0.0	0.5	0.8	0.1	0.0	0.5	0.5	0.0	0.0	0.0	0.0	0.0	0.7	0.0	0.0	0.0
**Myrcane**	0.0	6.1	0.7	0.0	0.0	0.0	0.0	0.0	0.0	0.0	0.0	0.0	5.8	5.7	0.0	0.0
**Pinane**	0.0	7.6	3.1	0.0	2.1	0.0	0.0	0.0	0.0	0.0	22.8	2.2	11.7	0.0	0.0	2.2
**Thujopsane**	0.0	0.0	0.0	0.0	1.9	0.0	0.0	0.0	0.0	0.0	0.0	0.0	0.0	0.0	0.0	0.0

Reference: RO (Morais et al. 2007); RS1 - 1 to 16 (Schindler et al. 2017); RS2 (Von Poser et al. 1994); RS3 (Péres et al. 2009); RS4 (Sperotto et al. 2013); SC (Chaaban et al. 2018); SP1- 1 and 2 (Andrade et al. 1998); PR1 (Krinski et al. 2016); PR2 (Bernuci et al. 2016); PR3 - 1 and 2 (Krinski et al. 2018); PR4 (Silva et al. 2019); PR5 - 1 and 2 (Quiqui et al. 2019); PR6 (Souza et al. 2020) and; PR7 (Souza et al. 2020).

Scheme S1. Biosynthetic map of terpene carbon skeleton types based on essential oils from leaves of from *Piper gaudichaudianum* Kunth of this study and database from literature.

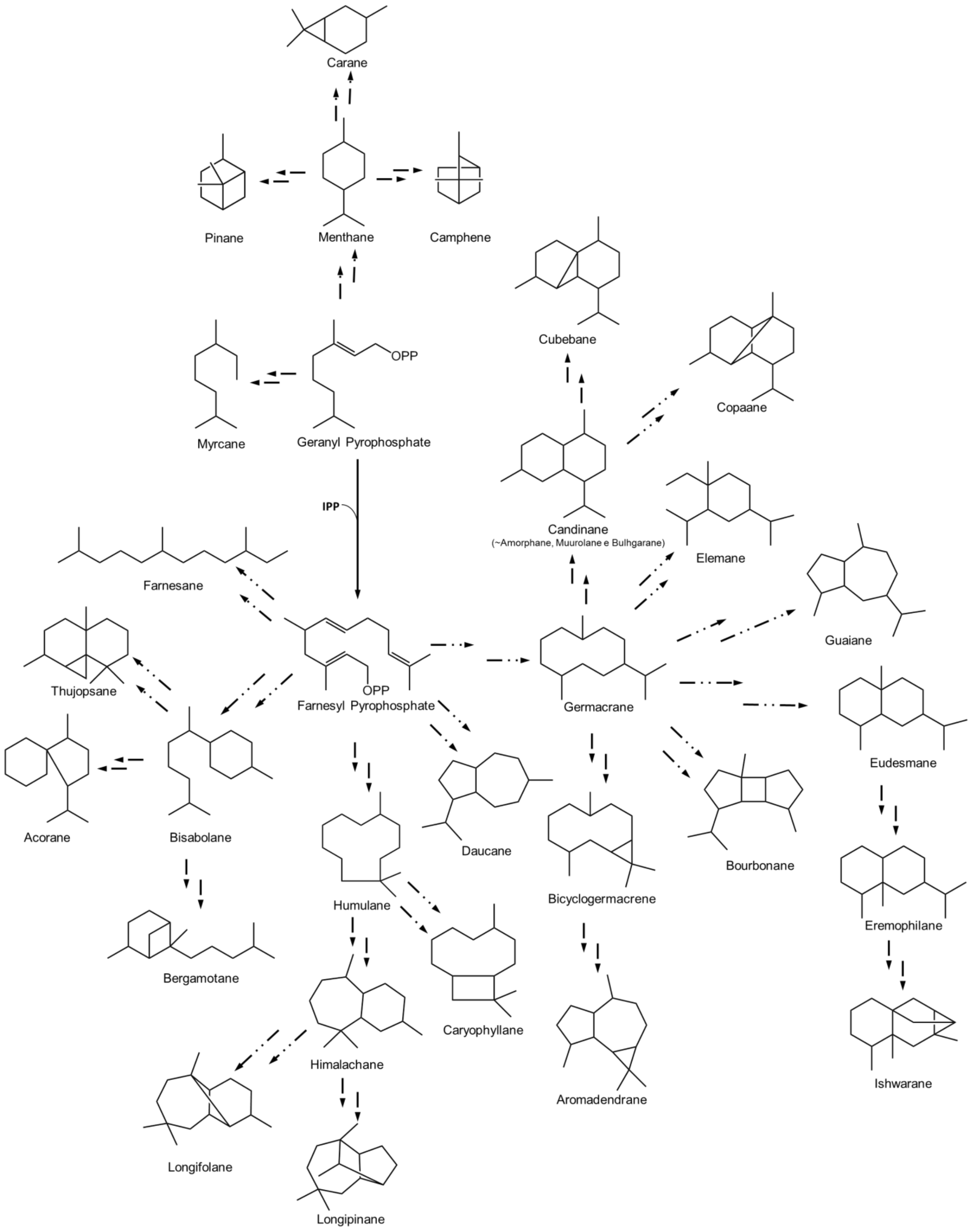

Reference: RO (Morais et al. 2007); RS1 - 1 to 16 (Schindler et al. 2017); RS2 (Von Poser et al. 1994); RS3 (Péres et al. 2009); RS4 (Sperotto et al. 2013); SC (Chaaban et al. 2018); SP1- 1 and 2 (Andrade et al. 1998); PR1 (Krinski et al. 2016); PR2 (Bernuci et al. 2016); PR3 - 1 and 2 (Krinski et al. 2018); PR4 (Silva et al. 2019); PR5 - 1 and 2 (Quiqui et al. 2019); PR6 (Souza et al. 2020) and; PR7 (Souza et al. 2020). Double arros indicate

that some steps are missed in the diagram. Broken line arrows represent a theoretical biosynthetic path that may or may not be active. Whole unbroken arrow represents a well established primary biosynthetic pathway.

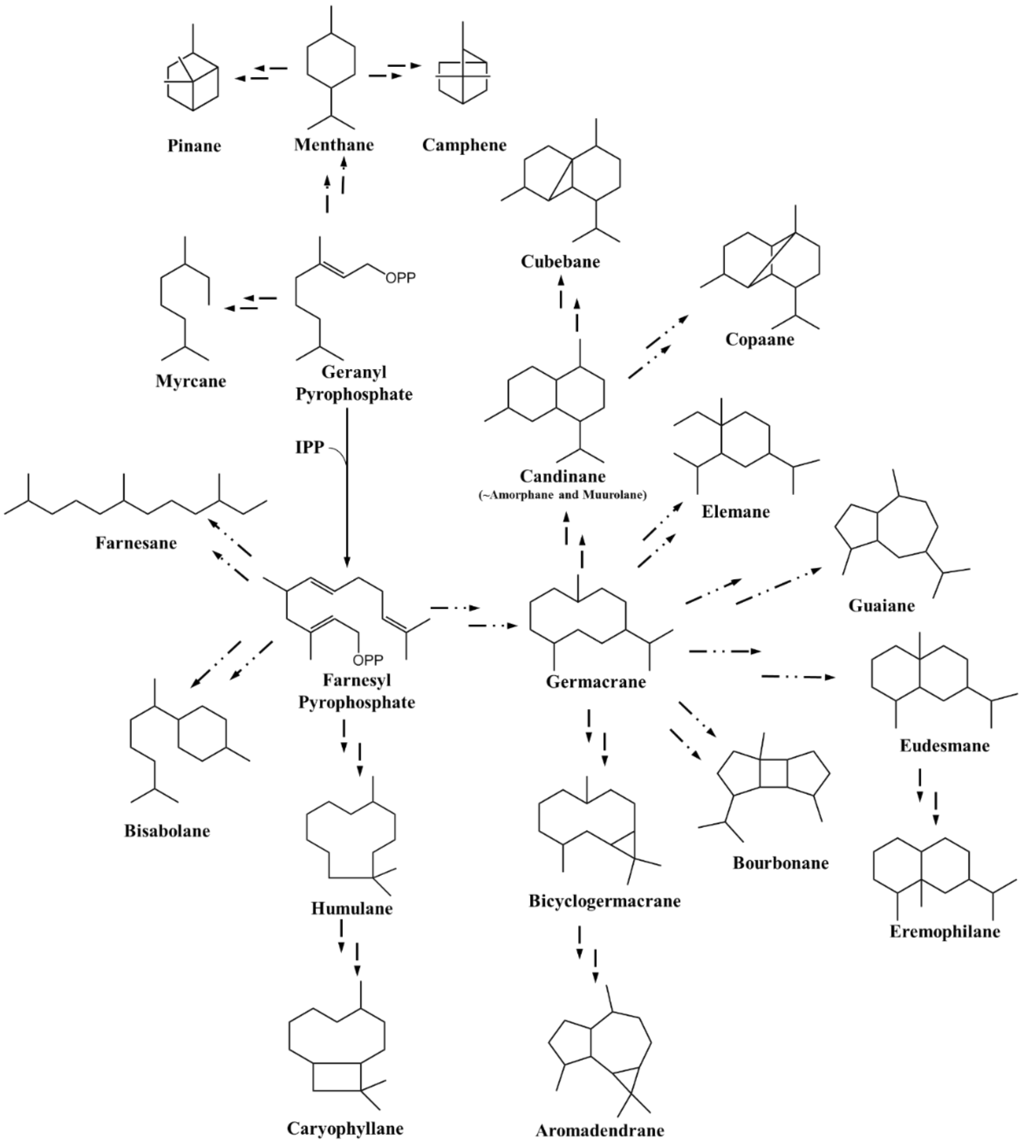

Scheme S2. Biosynthetic map of terpene carbon skeleton types based on the essential oils compounds from leaves of *Piper gaudichaudianum* Kunth (Piperaceae) for seasonality studies. Double arros indicate that some steps are missed in the diagram. Broken line arrows represent a theoretical biosynthetic path that may or may not be active. Whole unbroken arrow represents a well established primary biosynthetic pathway.

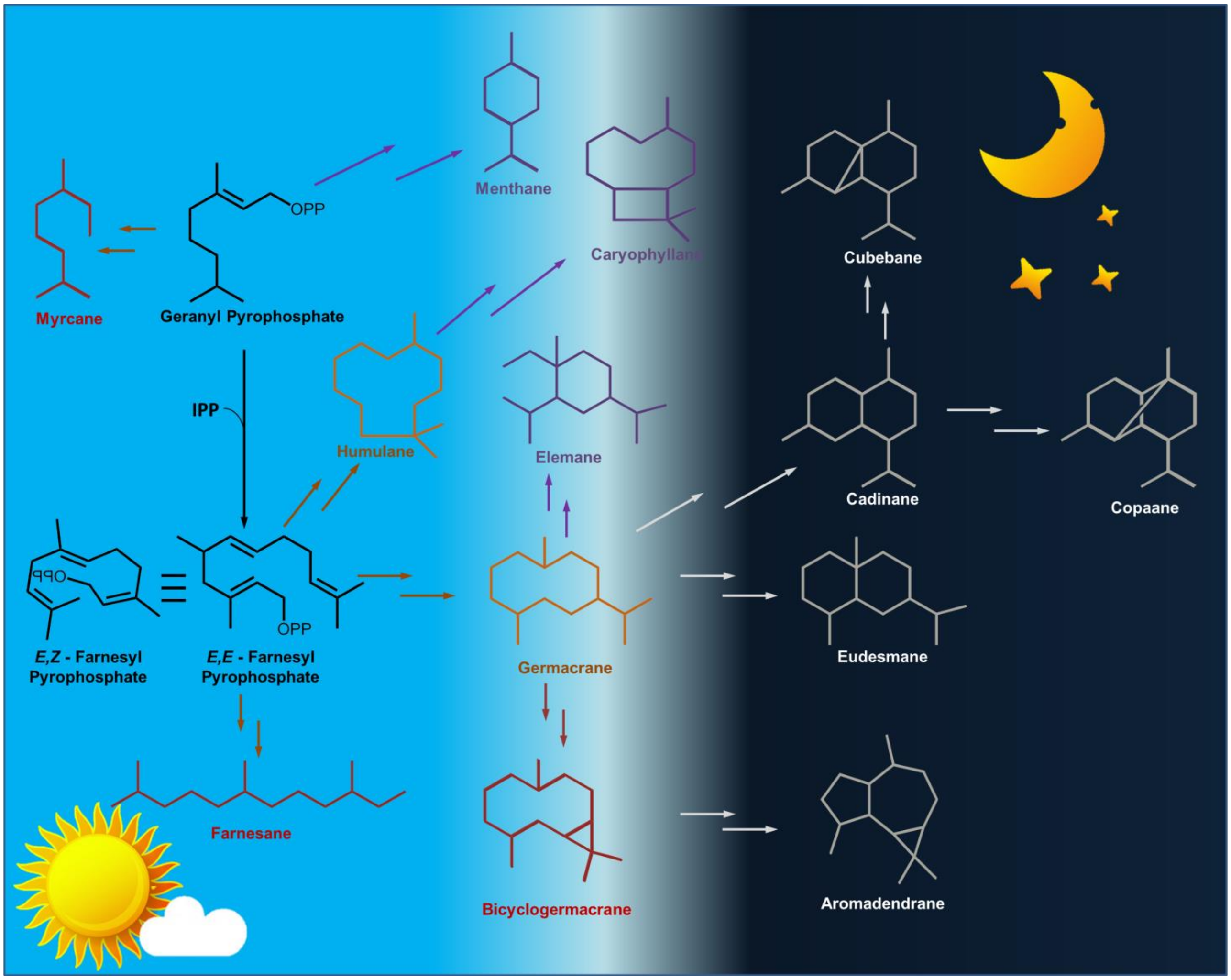

Scheme S3. Biosynthetic map of terpene carbon skeleton types based on the essential oils compounds from leaves of *Piper gaudichaudianum* Kunth (Piperaceae) for circadian rhythm studies.

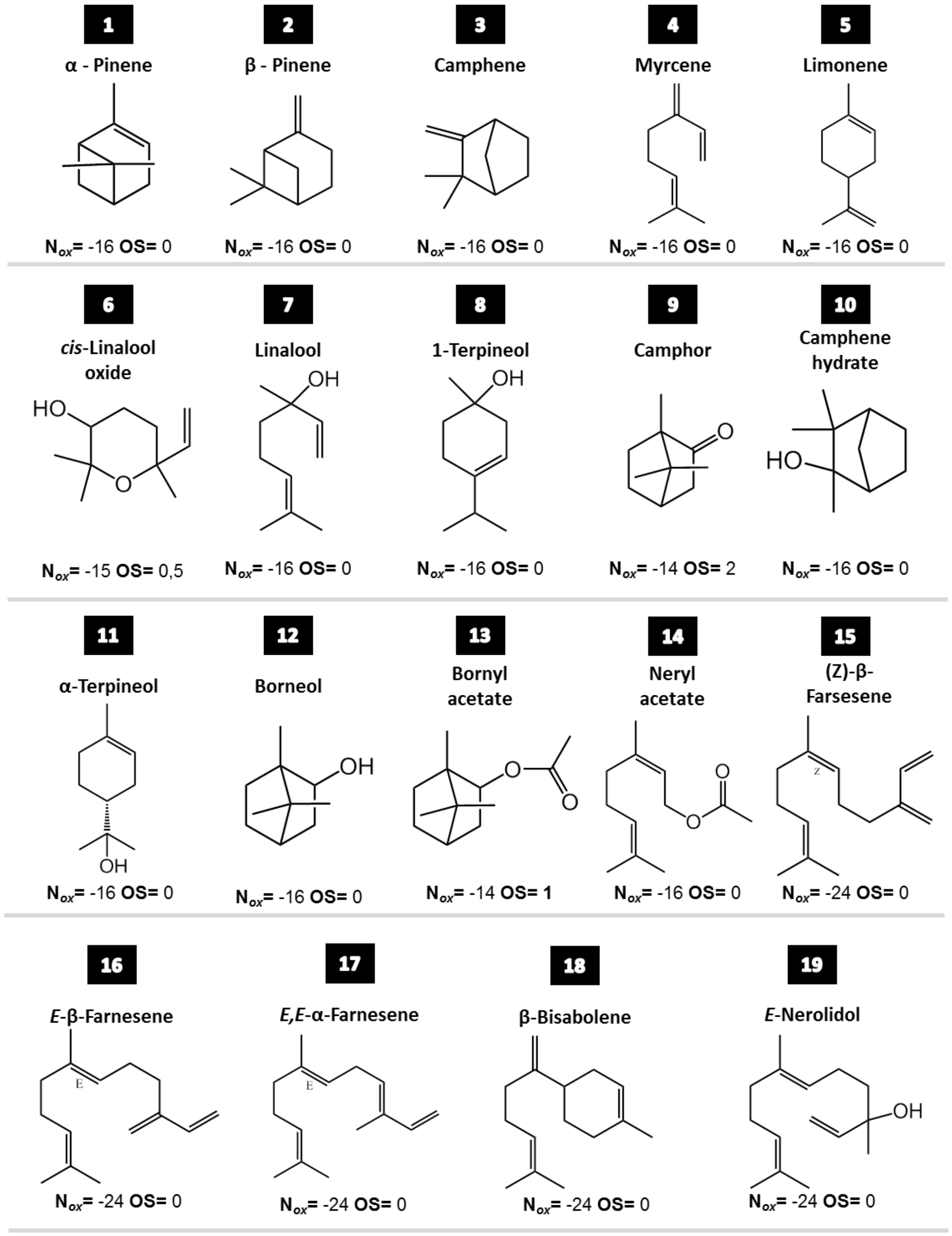

Figure S1. Continuation

20

***Z*-Nerolidol**

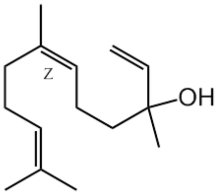

N_{ox}= -24 **OS=** 0

21

***iso*-Caryophyllene**

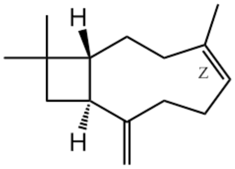

N_{ox}= -24 **OS=** 0

22

***E*-Caryophyllene**

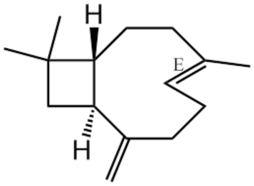

N_{ox}= -24 **OS=** 0

23

β-Humulene

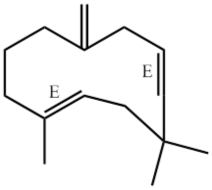

N_{ox}= -24 **OS=** 0

24

α-Humulene

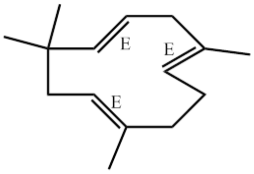

N_{ox}= -24 **OS=** 0

25

9-*epi*-*E*-Caryophyllene

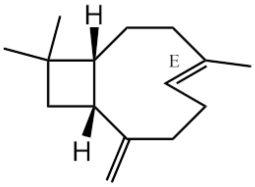

N_{ox}= -24 **OS=** 0

26

Humulene epoxide II

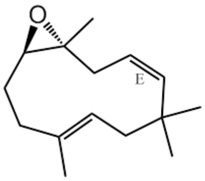

N_{ox}= -23 **OS=** 0,5

27

Caryophylla-4(12),8(13)-dien-5α-ol

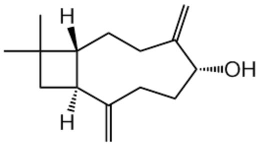

N_{ox}= -22 **OS=** 1

28

14-hydroxy-*Z*-Caryophyllene

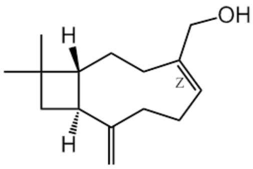

N_{ox}= -22 **OS=** 1

29

14-hydroxy-9-*epi*-*E*-Caryophyllene

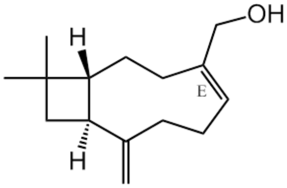

N_{ox}= -22 **OS=** 1

30

Caryophyllene acetate

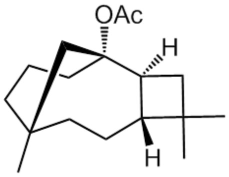

N_{ox}= -22 **OS=** 1

31

Caryophyllene oxide

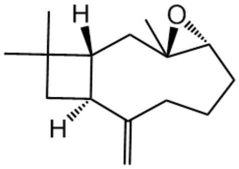

N_{ox}= -22 **OS=** 1

32

Germacrene D

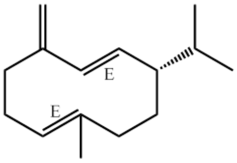

N_{ox}= -24 **OS=** 0

33

Germacrene B

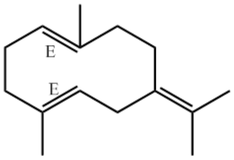

N_{ox}= -24 **OS=** 0

34

Bicyclogermacrene

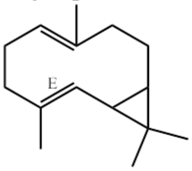

N_{ox}= -24 **OS=** 0

35

α-Gurjunene

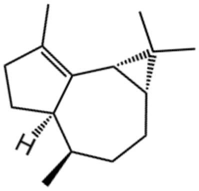

N_{ox}= -24 **OS=** 0

Figure S1. Continuation

36

β-Gurjunene

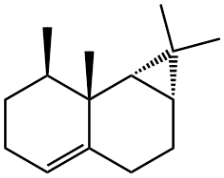

N$_{ox}$**=** -24 **OS=** 0

37

Aromadendrene

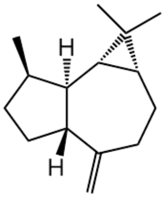

N$_{ox}$**=** -24 **OS=** 0

38

***allo*-Aromadendrene**

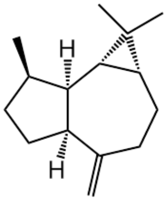

N$_{ox}$**=** -24 **OS=** 0

39

dehydro-Aromadendrane

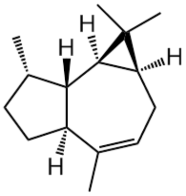

N$_{ox}$**=** -22 **OS=** 1

40

Spathulenol

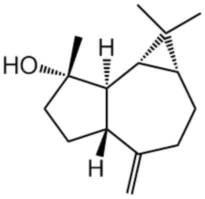

N$_{ox}$**=** -22 **OS=** 1

41

Viridiflorol

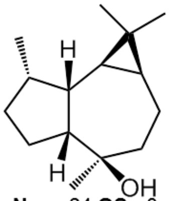

N$_{ox}$**=** -24 **OS=** 0

42

Ledol

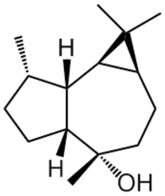

N$_{ox}$**=** -24 **OS=** 0

43

β-Bourbonene

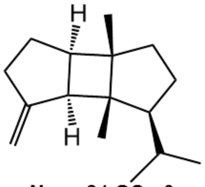

N$_{ox}$**=** -24 **OS=** 0

44

***cis*-Cadina-1(6),4-diene**

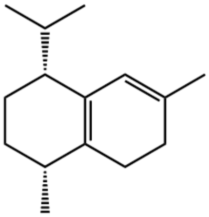

N$_{ox}$**=** -24 **OS=** 0

45

γ-Muurolene

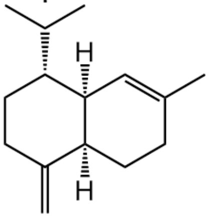

N$_{ox}$**=** -24 **OS=** 0

46

Amorpha-4,7(11)-diene

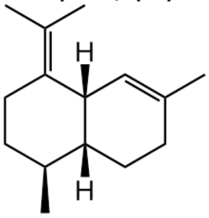

N$_{ox}$**=** -24 **OS=** 0

47

α-Amorphene

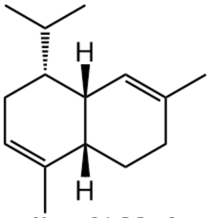

N$_{ox}$**=** -24 **OS=** 0

48

γ-Amorphene

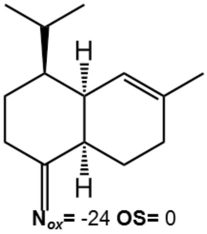

N$_{ox}$**=** -24 **OS=** 0

49

α-Muurolene

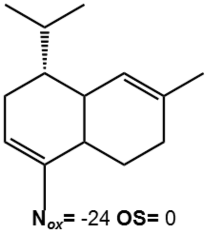

N$_{ox}$**=** -24 **OS=** 0

50

γ-Cadinene

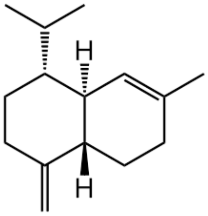

N$_{ox}$**=** -24 **OS=** 0

51

δ-Cadinene

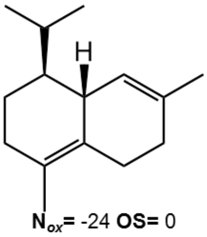

N$_{ox}$**=** -24 **OS=** 0

Figure S1. Continuation

52

Zonarene

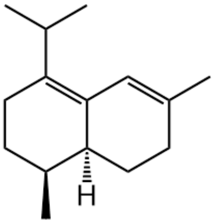

N_{ox}= -24 **OS=** 0

53

trans-Cadina-1,4-diene

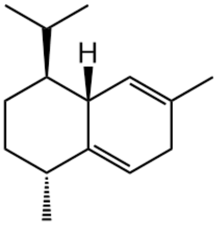

N_{ox}= -24 **OS=** 0

54

α-Cadinene

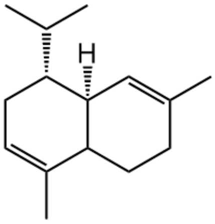

N_{ox}= -24 **OS=** 0

55

1,10-di-*epi*-Cubenol

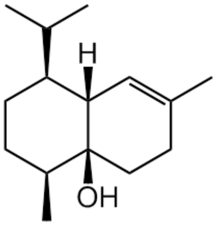

N_{ox}= -24 **OS=** 0

56

β-Calacorene

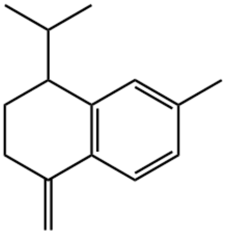

N_{ox}= -20 **OS=** 2

57

α-Corocalene

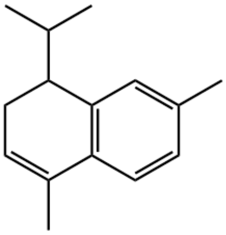

N_{ox}= -20 **OS=** 2

58

Muurola-4,10(14)-dien-1-β-ol

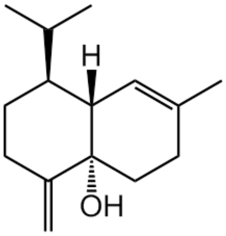

N_{ox}= -22 **OS=** 1

59

epi-α-Muurolol

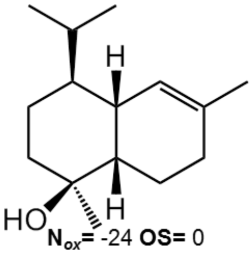

N_{ox}= -24 **OS=** 0

60

α-Muurolol

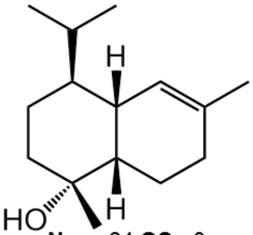

N_{ox}= -24 **OS=** 0

61

α-Cadinol

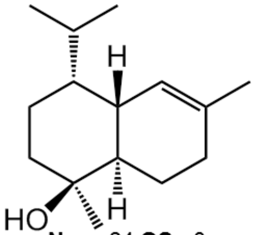

N_{ox}= -24 **OS=** 0

62

Cadalene

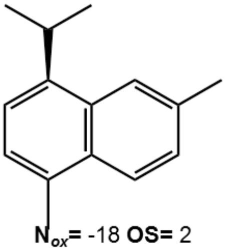

N_{ox}= -18 **OS=** 2

63

Amorpha-4,9-dien-2-ol

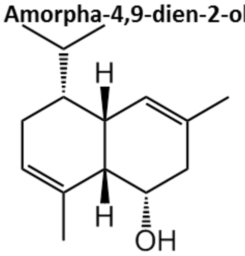

N_{ox}= -22 **OS=** 1

64

Amorpha-4,9-dien-14-al

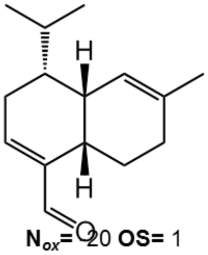

N_{ox}= -20 **OS=** 1

65

α-Cubebene

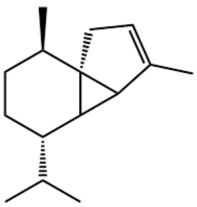

N_{ox}= -24 **OS=** 0

66

α-Ylangene

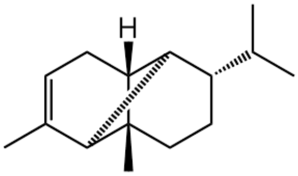

N_{ox}= -24 **OS=** 0

67

α-Copaene

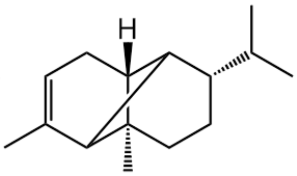

N_{ox}= -24 **OS=** 0

Figure S1. Continuation

68

β-Copaene

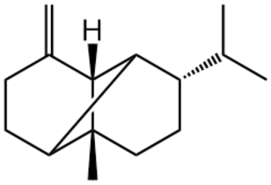

N_{ox}= -24 **OS=** 0

69

Bicycloelemene

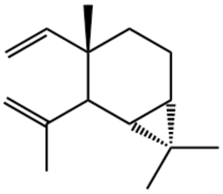

N_{ox}= -24 **OS=** 0

70

δ-Elemene

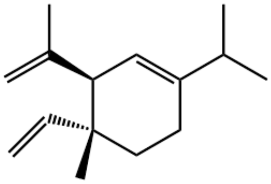

N_{ox}= -24 **OS=** 0

71

β-Elemene

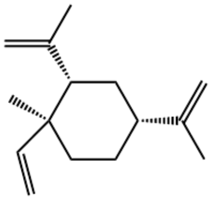

N_{ox}= -24 **OS=** 0

72

γ-Elemene

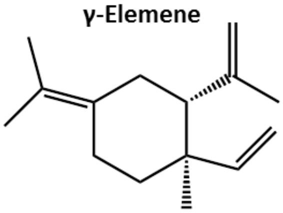

N_{ox}= -24 **OS=** 0

73

Aristolochene

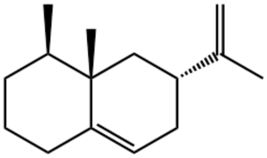

N_{ox}= -24 **OS=** 0

74

Valencene

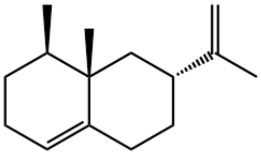

N_{ox}= -24 **OS=** 0

75

Elemol

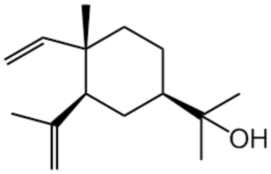

N_{ox}= -24 **OS=** 0

76

γ-Gurjunene

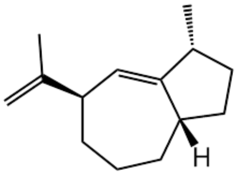

N_{ox}= -24 **OS=** 0

77

Eudesmadiene

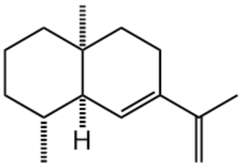

N_{ox}= -24 **OS=** 0

78

β-Selinene

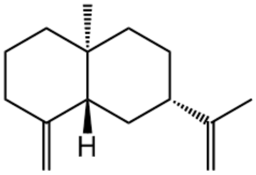

N_{ox}= -24 **OS=** 0

79

α-Selinene

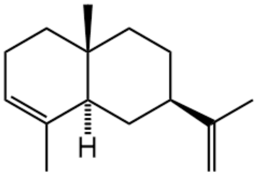

N_{ox}= -24 **OS=** 0

80

7-*epi*-α-Selinene

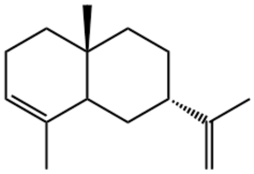

N_{ox}= -24 **OS=** 0

81

Selina-3,7(11)-diene

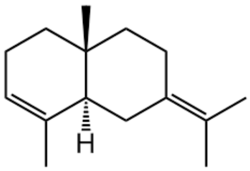

N_{ox}= -24 **OS=** 0

82

Rosifoliol

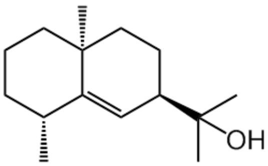

N_{ox}= -24 **OS=** 0

83

5-*epi*-7-*epi*-α-Eudesmol

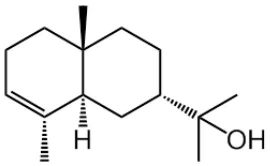

N_{ox}= -24 **OS=** 0

Figure S1. Continuation

84

γ-Eudesmol

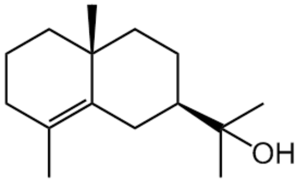

N_{ox}= -24 **OS=** 0

85

7-*epi*-α-Eudesmol

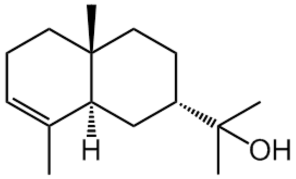

N_{ox}= -24 **OS=** 0

86

Selina-3,11-dien-6-α-ol

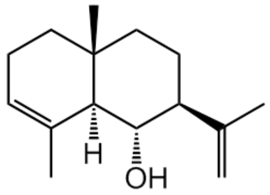

N_{ox}= -22 **OS=** 0

87

α-Eudesmol

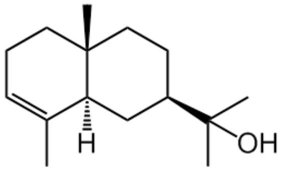

N_{ox}= -24 **OS=** 0

88

10-*epi*-γ-Eudesmol

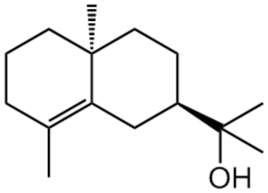

N_{ox}= -24 **OS=** 0

89

***cis*-Calamenen-10-ol**

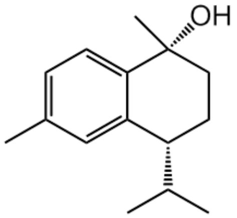

N_{ox}= -20 **OS=** 0

90

3*E*-Hexenol

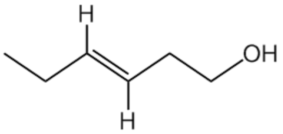

N_{ox}= -10 **OS=** 2

91

Nonanal

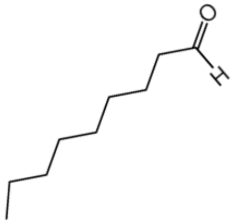

N_{ox}= -20 **OS=** 1

92

Geranyl acetate

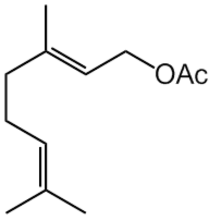

N_{ox}= -24 **OS=** 0

93

Undecanal

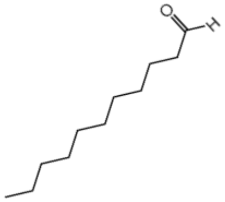

N_{ox}= -20 **OS=** 1

94

Octadecane

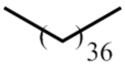

N_{ox}= -38 **OS=** 0

95

***cis*-ρ-Menth-2-en-1-ol**

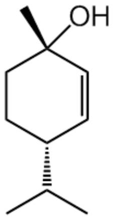

N_{ox}= -16 **OS=** 0

96

***cis*-ρ-Mentha-2,8-dien-1-ol**

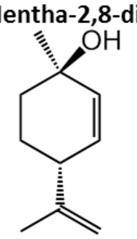

N_{ox}= -14 **OS=** 0

97

γ-Vetivenene

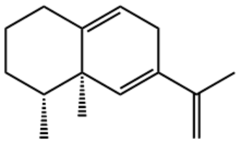

N_{ox}= -22 **OS= 1**

Figure S1. Structures of terpenic derivatives (monoterpenoids and sesquiterpenes) and non-terpenic

hydrocarbons identified in the essential oils of *Piper gaudichaudianum* Kunth with their respective oxidation numbers (N_{OX}) and the values of the oxidation steps (OS) of terpene-type chemical precursors.

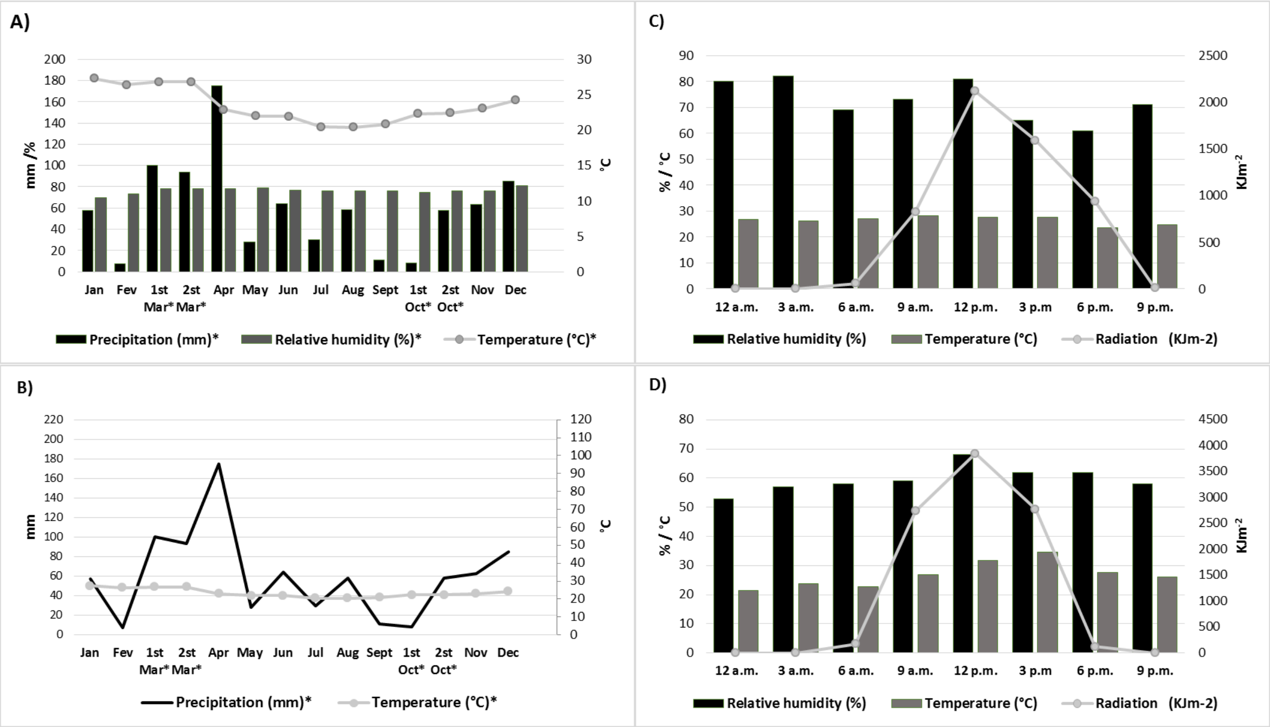

Figure S2. Climatic data of Rio de Janeiro City (Brazil) during the collections of *Piper gaudichaudianum* Kunth leaves. Data collected from reference INMET (2020). Monthly averages of temperature, precipitation, and relative humidity from January to December 2017 (A). Ombrothermal diagram from January to December 2017 (B). Data of temperature, relative humidity, and radiationfrom the leaves´ collection time for the circadian study in March (C) and October 2017 (D).
